# m6A hypomethylation of DNMT3B regulated by ALKBH5 promotes intervertebral disc degeneration via E4F1 deficiency

**DOI:** 10.1002/ctm2.765

**Published:** 2022-03-27

**Authors:** Gaocai Li, Rongjin Luo, Weifeng Zhang, Shujie He, Bingjin Wang, Huaizhen Liang, Yu Song, Wencan Ke, Yunsong Shi, Xiaobo Feng, Kangcheng Zhao, Xinghuo Wu, Yukun Zhang, Kun Wang, Cao Yang

**Affiliations:** ^1^ Department of Orthopaedics, Union Hospital, Tongji Medical College Huazhong University of Science and Technology Wuhan China; ^2^ Department of Cardiology, Union Hospital, and Key Laboratory of Biological Targeted Therapy of the Ministry of Education Tongji Medical College Huazhong University of Science and Technology Wuhan China

**Keywords:** ALKBH5, DNMT3B, intervertebral disc degeneration, m6A, nucleus pulposus cell senescence

## Abstract

**Background:**

The intervertebral disc (IVD) degeneration is the leading cause of low back pain, which accounts for a main cause of disability. *N*6‐methyladenosine (m6A) is the most abundant internal modification in eukaryotic messenger RNAs and is involved in various diseases and cellular processes by modulating mRNA fate. However, the critical role of m6A regulation in IVD degeneration remains unclear. Nucleus pulposus cell (NPC) senescence is critical for the progression of IVD degeneration. Here, we uncovered the role and explored the regulatory mechanism of m6A in NPC senescence during IVD degeneration.

**Methods:**

Identification of NPC senescence during IVD degeneration was based on the analysis of tissue samples and the cellular model. ALKBH5 upregulation inducing cellular senescence was confirmed by functional experiments in vivo and in vitro. ChIP‐qPCR and DNA‐Pulldown were used to reveal increased ALKBH5 was regulated by KDM4A‐mediated H3K9me3. Furthermore, Me‐RIP‐seq was performed to identify m6A hypomethylation of DNMT3B transcripts in senescent NPCs. Stability analysis showed that DNMT3B expression was enhanced for less YTHDF2 recognition and increased DNMT3B promoted NPC senescence and IVD degeneration via E4F1 methylation by in vivo and in vitro analyses.

**Results:**

Expression of ALKBH5 is enhanced during IVD degeneration and NPC senescence, due to decreased KDM4A‐mediated H3K9me3 modification. Functionally, ALKBH5 causes NPC senescence by demethylating DNMT3B transcripts and in turn promoting its expression via less YTHDF2 recognition and following degradation due to transcript hypomethylation in vitro and in vivo. Increased DNMT3B promotes the development of IVD degeneration and NPC senescence, mechanistically by methylating CpG islands of E4F1 at the promoter region and thus restraining its transcription and expression.

**Conclusions:**

Collectively, our findings reveal an epigenetic interplay mechanism in NPC senescence and IVD degeneration, presenting a critical pro‐senescence role of ALKBH5 and m6A hypomethylation, highlighting the therapeutic potential of targeting the m6A/DNMT3B/E4F1 axis for treating IVD degeneration.

## Graphical abstract

- Expression of ALKBH5 is enhanced during NPC senescence due to decreased KDM4A‐mediated H3K9me3 modification.
- ALKBH5 causes NPC senescence by demethylating DNMT3B transcripts and in turn promoting its expression via less YTHDF2 recognition and following degradation due to transcript hypomethylation.
- DNMT3B promotes IVD degeneration and NPC senescence by methylating E4F1 at the promoter and thus restraining its transcription and expression.

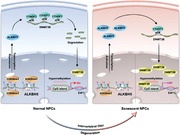

## 1 INTRODUCTION

The intervertebral disc (IVD) degeneration is the leading cause of low back pain and neck pain which account for tremendous societal and economic burdens for around 80% of the population worldwide.^1^ IVD is composed of inner nucleus pulposus (NP) tissue and outer circumferential annulus fibrosus and lies between two adjacent vertebral bodies to cushion loads and facilitate body movements. The degeneration of IVD is characterized with lose of intervertebral space height and reduced hydration of NP due to proteoglycans loss and dysfunction of nucleus pulposus cells (NPCs).^2^ However, under stimulation of various harmful factors including aging, trauma and excessive loading, the pathogenesis of IVD degeneration is complex and worthy of further investigation and elucidation.

NP is the core compartment of IVD and functions critically in the structure and stability of the disc, and thus the degeneration of NP especially the degenerative change of NPCs accounts for the major pathogenesis of this process.^3^, ^4^, ^5^ Accumulating evidence indicates that the senescence of NPCs is the leading factor that causes the loss of number and function of NPCs, which has paved a novel prospect for the prevention and cure of IVD degeneration.^6^, ^7^, ^8^ Under multiple long‐term stimuli of extrinsic and intronic factors with aging, NPCs gradually decrease in function and get senescent, which may result in the change of the disc microenvironment, decrease of extracellular matrix and downregulation of growth factors.^9^, ^10^ However, the specific mechanism of NPC senescence during the process of IVD degeneration remains to be further elucidated.

Aging‐associated disorders of multiple tissues and organs are accompanied with altered epigenetic mechanisms, including DNA methylation, non‐coding RNAs, chromatin remodeling and histone modification.^11^ The DNA methylation levels are altered during aging, which could be used to predict chronological aging status of a variety of organs.^12^, ^13^ Recent studies show non‐coding RNAs, including lincRNAs, microRNAs and circRNAs, provide additional layers of epigenetic regulation that are important in the context of aging.^14^, ^15^, ^16^, ^17^ Furthermore, modifications of histone and chromatin accessibility regulation are also demonstrated with critical function to modulate aging kinetics.^18^, ^19^, ^20^ However, in the epigenetic field, whether NPC cellular senescence could be regulated at histone‐modification or post‐transcriptional level remains unknown.

*N*6‐methyladenosine (m6A), one chemical methylation modification of RNA, is the most abundant internal modification in eukaryotic messenger RNAs, and accumulating evidences indicate fundamental regulatory roles of m6A modification in a variety of biological and pathological processes.^21^, ^22^, ^23^, ^24^ The modification level of m6A is regulated by the balanced activity and expression of writer and eraser proteins. The addition of a methyl group to the N6 site of adenine in the sequence motif of RRACU (R refers to G or A) is catalysed by methyltransferase complex composed of methyltransferase 3 (METTL3), methyltransferase 14 (METTL14) and WT1 associated protein (WTAP), while demethylation protein FTO alpha‐ketoglutarate dependent dioxygenase (FTO) and alkB homolog 5 (ALKBH5) could inverse this process.^25^, ^26^ Moreover, a group of specific reader proteins such as YTHDF family, IGF2BPs or YTHDC1, could be recruited to the m6A sites and execute regulatory function after recognition.^27^, ^28^, ^29^, ^30^ Binding YTHDF2 would target m6A‐containing RNAs to cytoplasmic decay sites for degradation while IGF2BPs’ recognition could stabilize the mRNAs and promote the translation of the targets.^27^ Furthermore, the splicing process and nucleus exportation of methylated transcripts could be affected by YTHDC1.^29^, ^31^ Recently, Hirayama et al. showed that FTO, the demethylase for m6A modification, regulated G1 cell‐cycle progression by targeting cyclin D1 mRNA.^32^ What's more, in the pathogenesis of heart failure with aging, METTL3‐mediated methylation of mRNA could lead to cardiomyocyte hypertrophy, thus diminishing cardiac homeostasis and function.^33^ Given the fact that epigenetic alterations contribute immensely to cell cycle progression, we reasoned that RNA modifications might also represent a distinct layer of epigenetic regulation in the senescence of NPCs.

In this study, we revealed that NPC aging is accompanied with higher expression of ALKBH5, which is one demethylase of m6A modification, in both degenerated NP tissues and cultured senescent NPCs due to epigenetic decrease of H3K9me3 modification in the promoter. Furthermore, loss of m6A modification of DNMT3B mRNA with aging results in elevation of DNMT3B via decreased YTHDF2‐mediated transcript decay, which further leads to hypermethylation of E4F1 at the DNA promoter region and less expression of E4F1. Moreover, silencing of ALKBH5 or DNMT3B in vivo could stall injury‐induced IVD degeneration to some extent. Additionally, gain and loss of function investigations demonstrated E4F1 insufficiency could result in NPC senescence and overexpression of E4F1 could rescue the pro‐senescence effect of ALKBH5 or DNMT3B to some extent in NPC aging. Altogether, our data revealed, under the epigenetic regulation of H3K9me3, ALKBH5‐mediated RNA demethylation could modulate the DNA methylation level of E4F1, and hence affect NPC senescence and IVD degeneration. Collectively, our study clarified the crosstalk of methylation regulation from different levels, including histone modification, m6A and DNA methylation during cellular senescence.

## 2 MATERIALS AND METHODS

### 2.1 Clinical samples collection

NP tissues were obtained from 52 patients (29 females and 23 males; aged 55.5 ± 3.55 years; Grades IV (*n* = 35) and V (*n* = 17)) with degenerative disc diseases undergoing surgery. The control samples were taken from 43 patients (26 females and 17 males; aged 15.5 ± 1.68 years; Grade I (*n* = 23) and II (*n* = 20) undergoing surgery due to scoliosis or thoracolumbar fracture after informed consent was obtained. The specimen information was provided in Table S4. All specimens were obtained from lumbar discs. Randomly selected normal (*n* = 16) and degenerative (*n* = 16) samples were used to perform in vivo tissue analysis (5 normal and 5 degenerative samples for RNA Scop; 5 normal and 5 degenerative samples for IHC; 6 normal and 6 degenerative samples for western blot). The degenerative changes of the IVDs were evaluated according to the Pfirrmann grade of patients’ magnetic resonance images.^8^ This study protocol was approved by the Ethics Committee of Tongji Medical College, Huazhong University of Science and Technology (No. S341).

### 2.2 NP cell isolation and culture

The NP tissues were cut into pieces after collection during surgery, and collagenase type II (Invitrogen, Carlsbad, CA, USA) was used to digest for 8 h at 37°C at the concentration of 0.2%. Then, cells from the digest were collected by centrifuging at 1200 rpm and then was cultured in DMEM (Gibco, Grand Island, NY, USA) supplied with 10% Fetal Bovine Serum （FBS) (Invitrogen), 1% penicillin–streptomycin (Sigma), 2 mM glutamine (Sigma), and 50 μg/ml l‐ascorbic acid (Sigma) at 37°C in 5% CO_2_. When grown to confluence, 0.25% trypsin/1 mM EDTA was used to digest the cells for passing for expansion. Passage 2 cells were plated into experimental plates or bottles for following experiments. In some experiments, cells were stimulated by 20 ng/ml of TNF‐α (Peprotech, 300–01A, ROCKY HILL, New Jersey) in the culture medium for 48 h. Unstimulated cells were used as controls.

### 2.3 Western blot

Samples were lysed by RIPA (Beyotime, P0013B, Shanghai, China) and the Micro BCA Protein Assay Kit (Beyotime, P0010S, Shanghai, China) was used to measure the protein content. Sodium dodecyl sulfate (SDS)‐polyacrylamide gel electrophoresis gels were used for electrophoresis and then transferred to polyvinylidene difluoride (PVDF) membranes. Then, blocking buffer was used to block for 1 h and following the specific primary antibodies (the primary antibodies used were listed in Table S1) were used to incubate with the membranes overnight at 4°C. Then, diluted horseradish peroxidase (HRP)‐conjugated Affinipure Goat Anti‐Rabbit IgG (SA00001‐2, Proteintech) and HRP‐conjugated Affinipure Goat Anti‐Mouse IgG (SA00001‐1, Proteintech) using antibody dilution were used to incubate the membranes. And GAPDH or H3 was used a loading protein control. At last, enhanced chemiluminescence reagents (Affinity, KF001, Nanjing, China) used to visualize the protein expression using the ChemiDoc MP Imaging System (Bio‐Rad, 12003154 Hercules, CA, USA). And ImageJ was used for semi‐quantification of the expression of proteins.

### 2.4 RNA‐seq

NPCs were isolated from specimens collected during surgery using collagenase. NPCs from four normal IVD samples were considered normal and NPCs from degenerated samples were considered senescent NPCs. Then TRIzol™ Reagent (Thermo Fisher, 15596026) was used to extract RNA. Then, library preparation and sequencing were conducted using Illumina HiSeq 2000 and analysis was carried out by Biocame. The NGS data of NPCs transcript in this study are available under the accession identifier GSE167931.

### 2.5 Me‐RIP‐seq

Cultured NPCs at passage 2 were treated with TNFα for 48h or not with three samples for each group, followed by RNA extraction using TRIzol™ Reagent (Thermo Fisher, 15596026). Then, Dynabeads® mRNA purification kit (Invitrogen) was used to isolated polyadenylated RNA from total RNA. On the basis of previously published protocols (https://doi.org/10.1038/nature11112.), we performed RNA fragmentation, Me‐RIP and library preparation. Sequencing was conducted using Illumina NovaSeq 6000 and analysis was carried out by Epibiotek. The raw data from the Me‐RIP‐Seq analysis of NPCs were deposited in the Gene Expression Omnibus database under the accession code GEO: GSE169484.

### 2.6 RNA scope

RNA scope was performed according to RNA scope Fluorescent Multiplex kit instructions (Advanced Cell Diagnostics, Hayward, CA). Single RNA molecules can be detected by way of its zz oligo pair design and DNA‐based amplification methods (https://doi.org/10.1016/j.jmoldx.2011.08.002). The DNMT3B probe was designed against nucleotides 1379‐2861. Slides were washed with 10% formamide in 2X SSC and staining with 4',6‐diamidino‐2‐phenylindole (DAPI). Then, we captured the images using microscope.

### 2.7 β‐galactosidase staining by immunofluorescence (IF) for NPCs

After corresponding treatment of TNFα or not, β‐galactosidase substrate C_12_FDG (Fluorescein di‐B‐D‐galactopyranose) was used to incubate NPCs at the concentration of 33 mM in 2 ml medium for 2 h, pretreated with 100 nM bafilomycin A1 for 1 h at 37°C. After being washed with PBS, NP cells were fixed with 4% paraformaldehyde for 15 min at room temperature. Then, 0.1 g/ml DAPI (Beyotime, Shanghai, China) was used to co‐stain the nucleus. This was followed by visualization and capturing of the images under a microscope. The experiments were replicated three times.

### 2.8 EdU incorperation assay

EdU labelling was performed to examine the proliferation status of NPCs. NPCs were exposed to 25×10^−6^ M of 5‐ethynyl‐2′‐deoxyuridine (EdU, RiboBio, C10338, Guangzhou, China) for 2 h at 37°C and fixed in 4% paraformaldehyde. NPCs were then permeabilised using 0.5% Triton‐X‐100 and then reacted with Apollo488 for 30 min. Subsequently, Hoechst 33342 was used to stain the DNA contents of the cells for 30 min, and images were visualized and captured using a microscope (Olympus, BX53). EdU positive cells were analysed using Image J. The experiments were replicated three times.

### 2.9 RNA‐pulldown

The biotinylated DNA probes containing T7 and SP6 promoter complementary to DNMT3B (Table S2) were synthesized and dissolved in 500 μl of lysis buffer (0.5 M NaCl, 20 mM Tris‐HCl, pH 7.5, and 1 mM EDTA). Then, following steps of RNA‐pulldown experiments were performed according to the manufacturer's instructions of a MagCapture™ RNA Pull Down kit (Millipore Corporation, USA). The cell lysates were incubated with probe‐coated beads. The pull‐down mixture was then used for following western blot analysis.

### 2.10 Subcellular fractionation for RT‐qPCR

RNA extraction was performed using PARIS™ kit (Invitrogen™: AM1921) and real‐time PCR were performed according to the protocol of instructions, GAPDH was used as endogenous control for the cytoplasmic RNA, while 18S RNA was selected as endogenous control for the nuclear RNA.

### 2.11 Statistical analysis

Data are presented as the mean ± SD of at least three independent experiments. GraphPad Prism 8 software (La Jolla, CA, USA) was used for statistical analysis. Two‐tailed unpaired Student's *t*‐test was used to measure the statistical significance of the difference. *P* < 0.05 was considered statistically significant while *P *> 0.05 was considered none significant (ns) (^#^ *P *> 0.05, *P < 0.05, **P < 0.01, ***P < 0.001 and ****P < 0.0001).

More Materials and Methods are available in Supporting information.

## 3 RESULTS

### 3.1 ALKBH5‐mediated mRNA demethylation contributes to NPC senescence

**FIGURE 1:**
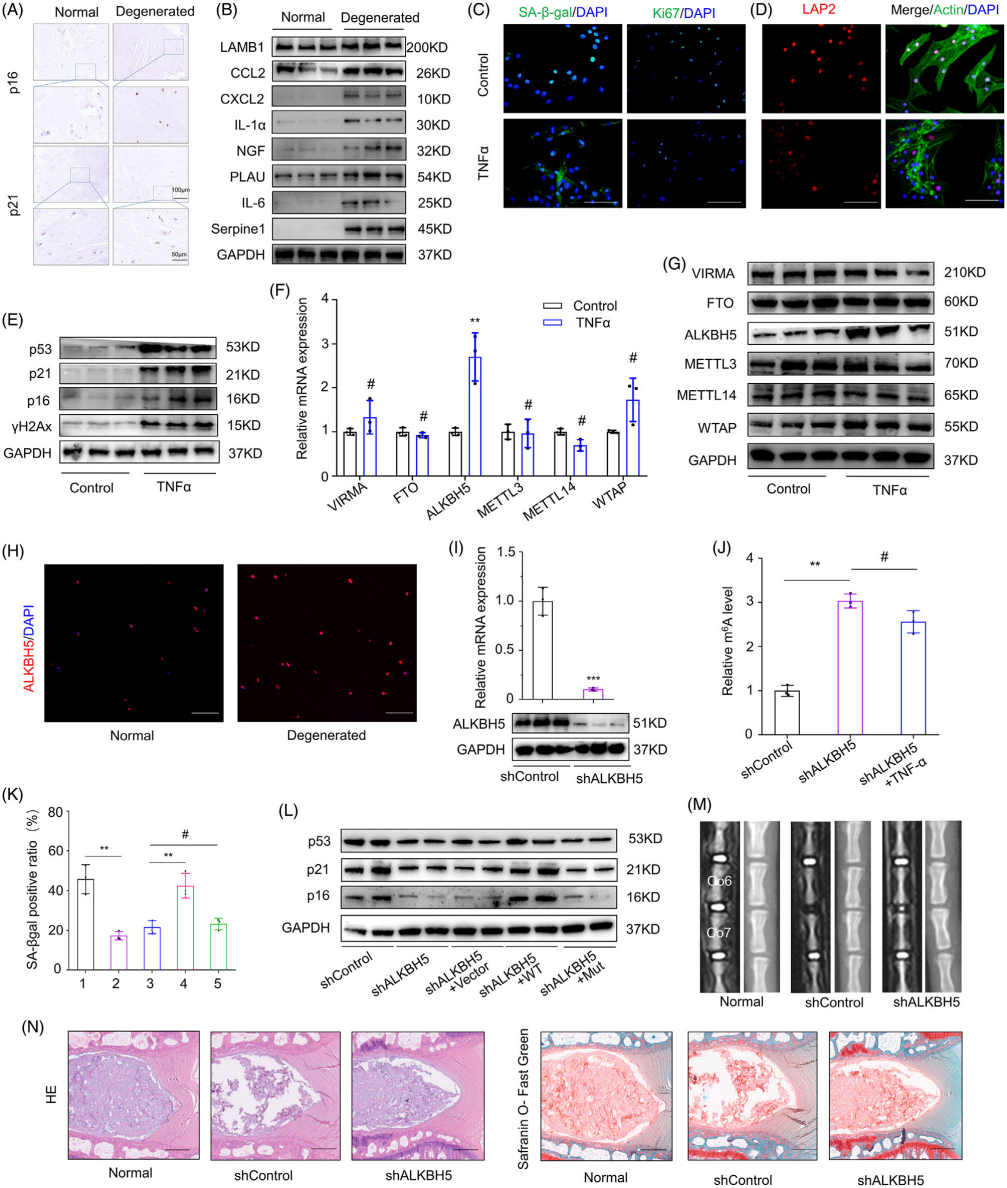
ALKBH5‐mediated mRNA demethylation contributes to nucleus pulposus cell (NPC) senescence. (A) IHC analysis of p16 and p21 expression in normal and degenerated NP tissues (scale bar: 100 μm, 50 μm). (B) Protein level analysis of senescence‐associated secretory phenotype (SASP) and senescence indicators in normal and degenerated NP tissues by western blot; GAPDH was used as a loading control (*n* = 3). (C) IF analysis of SA‐β‐gal activity (green: SA‐β‐gal; blue: DAPI; scale bar: 50 μm) and Ki67 expression (green: SA‐β‐gal; blue: DAPI; scale bar: 100 μm) in normal and senescent NPCs. (D) IF analysis of LAP2 expression (red: LAP2; blue: DAPI; green: F‐actin; scale bar: 50 μm) in normal and senescent NPCs. (E) Protein level analysis of senescence indicators in normal and senescent NPCs by western blot, GAPDH was used as a loading control. (F) Protein level analysis of methyltransferases and demethylases in normal and senescent NPCs by western blot, GAPDH was used as a loading control. (G) mRNA level analysis of methyltransferases and demethylases in normal and senescent NPCs by RT‐qPCR, GAPDH was used as a loading control, *n* = 3. **P < 0.01, ^#^ *P *> 0.05, two‐tailed unpaired Student's *t*‐test. (H) IF analysis of ALKBH5 expression in normal and degenerated NP tissues (red: ALKBH5; blue: DAPI; scale bar: 100 μm). (I) Knockdown efficiency of ALKBH5 in NP cells by shRNA, analysed by western blot and RT‐qPCR, GAPDH was used as a loading control, *n* = 3. ***P < 0.001, two‐tailed unpaired Student's *t*‐test. (J) Quantification of m6A modification level using ELISA‐based m6A colorimetric assay, *n* = 3. ^#^ *P *> 0.05, **P < 0.01, two‐tailed unpaired Student's *t*‐test. (K) SA‐β‐gal positive ratio of NPCs with ALKBH5 silencing accompanied with overexpression of wild‐type or mutant ALKBH5 (groups of 1, 2, 3, 4, 5 represent shContorl, shALKBH5, shALKBH5+Vector, shALKBH5+WT, shALKBH5+Mut), *n* = 3. **P < 0.01, two‐tailed unpaired Student's *t*‐test. (L) Protein level analysis of senescence indicators in NPCs by western blot, GAPDH was used as a loading control. (M, N) Radiographic imaging analysis and histological staining of a rat model of intervertebral disc (IVD) degeneration (scale bar: 500 μm) with or without ALKBH5 silencing

The relationship between disc degeneration and NP cellular senescence has been widely reported and to explore the aging status of NPCs in the degeneration process of IVDs, we verified the expression of aging‐associated markers p16 and p21 by immunohistochemistry (IHC) in NP tissues with various degrees of disc degeneration (Figure 1A). Furthermore, increased levels of represent senescence‐associated secretory phenotype (SASP) and senescence indicators were detected by western blot in NPCs from normal and degenerated discs, indicating NPC cellular senescence occurred during IVD degeneration (Figure 1B). To further explore the underlying pathological mechanism of NPC senescence, we isolated human NPCs from normal and degenerated discs to perform RNA sequencing, and gene set enrichment analysis (GSEA) results showed that degeneration of NP was accompanied with NPC senescence (Figure S1A). Subsequently, TNFα administration was performed to establish the degenerated in vitro model of NPCs. And the treatment of TNFα could induce senescence of NPCs according to increased SA‐β‐gal activity and decreased proportion of Ki67‐positive cells (Figure 1C). Meanwhile, western blot and immunofluorescence analysis of representative senescence markers verified the former results (Figure 1D, E and Figure S1B).

To study the role of m6A modification in IVD degeneration and NPC senescence, we examined the expression levels of methyltransferases and demethylases, the main regulators of m6A modification, in the NPCs by western blot and RT‐qPCR, and identified that ALKBH5, the core component of demethylase, was increased in senescent NPCs (Figure 1F, G and Figure S1C). Consistent with our findings, the expression of ALKBH5 was found significantly up‐regulated in the transcript sequencing data of NPCs from degenerated and normal discs, which was further confirmed by immunofluorescence staining of NP tissues (Figure S1D and Figure 1H). To investigate the functional roles of ALKBH5 and m6A modification in NPC senescence, we silenced ALKBH5 in NPCs using lentivirus‐based shRNA, and the knockdown efficiency was verified by western blot analysis and RT‐qPCR (Figure 1I). The higher level of m6A modification in NPCs was observed in ALKBH5 knockdown NPCs, though treated with TNFα, indicated by Elisa‐based m6A colorimetric assay (Figure 1J). We evaluated the aging‐related characteristics of NPCs after ALKBH5 silencing, and found expression of senescence markers and SA‐β‐gal activity were decreased compared with wild type NPCs treated by TNFα (Figure S1E and Figure 1K). Furthermore, overexpression of ALKBH5 in ALKBH5‐silenced NPCs attenuated senescent phenotype to some extent, while overexpression of mutant ALKBH5 disrupted with enzymatic activity by carrying the H204A mutation failed to rescue senescent phenotype^34^ (Figure 1L), indicating that the enzymatic activity of ALKBH5 is required for cellular senescence. Besides, we established a rat model of IVD degeneration using the needle puncture and injected into the IVD using a 29‐gauge fine needle with injection lentivirus containing shRNA against ALKBH5 or vector to testify the role of ALKBH5 in vivo. First, transcript expression analysis of ALKBH5 by RT‐qPCR in Rat NP tissue reflects the silencing efficiency (Figure S1F). The radiographic imaging analysis combined with histological staining indicated intervention of ALKBH5 could ameliorate the aging and degeneration of IVD (Figure 1M, N and Figure S1G, H). These results suggest ALKBH5‐mediated lower level of m6A modification contributes a lot to the senescence of NPCs and IVD degeneration.

### 3.2 ALKBH5 is regulated by epigenetic alteration of H3K9me3

**FIGURE 2:**
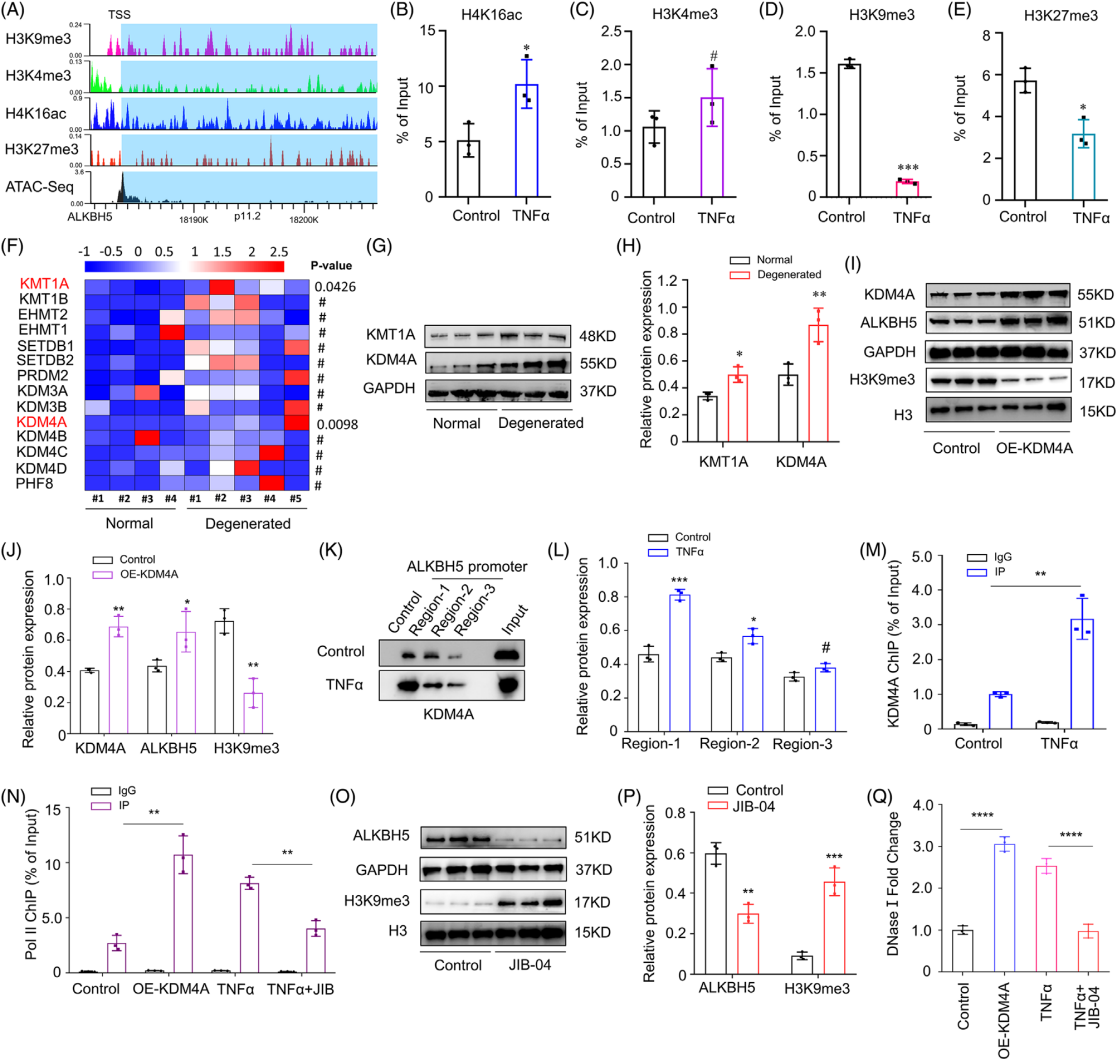
ALKBH5 is regulated by epigenetic alteration of H3K9me3. (A) Prediction of four types of histone modification and ATAC‐seq of the ALKBH5 promoter by the online tool ENCODE, the vertical axis represents signal intensity. (B–E) ChIP‐qPCR of H4K16ac, H3K4me3, H3K9me3, and H3K27me3 of the promoter of ALKBH5, *n* = 3. *P < 0.05, ***P < 0.001, ^#^ *P *> 0.05, two‐tailed unpaired Student's *t*‐test. (F) Heatmap of transcriptome sequencing data of methyltransferases and demethylases in normal and senescent NPCs. (G, H) Protein expression of KMT1A and KDM4A in normal and degenerated NP tissues by western blot analysis, GAPDH was used as a loading control, *n* = 3. *P < 0.05, **P < 0.01, two‐tailed unpaired Student's *t*‐test. (I, J) Expression of KDM4A, ALKBH5 and H3K9me3 in KDM4A‐overexpressing NPCs by western blot analysis; GAPDH or H3 was used as a loading control, *n* = 3. *P < 0.05, **P < 0.01, two‐tailed unpaired Student's *t*‐test. (K, L) Immunoblot validation and analysis of DNA pull‐down assay showing increased binding of KDM4A to the ALKBH5 promoter in senescent NPCs. (M) ChIP‐qPCR analysis of KDM4A enrichment with ALKBH5 at the promoter region in normal or senescent NPCs; IgG was used as a negative control, *n* = 3. **P < 0.01, two‐tailed unpaired Student's *t*‐test. (N) ChIP‐qPCR analysis of Pol II recruitment to the ALKBH5 promoter region in normal or senescent NPCs; IgG was used as a negative control, *n* = 3. ***P < 0.001, ****P < 0.0001, two‐tailed unpaired Student's *t*‐test. (O, P) Expression of ALKBH5 and H3K9me3 in NPCs with or without the H3K9me3 demethylase inhibitor JIB‐04, analysed by western blot; GAPDH or H3 was used as a loading control, *n* = 3. *P < 0.05, two‐tailed unpaired Student's *t*‐test. (Q) Chromatin accessibility of the ALKBH5 promoter in KDM4A‐overexpressing and TNFα‐induced senescent NPCs analysed by DNase I sensitivity assay, *n* = 3. ****P < 0.0001, two‐tailed unpaired Student's *t*‐test

Histone modification is another widely regulatory mechanism that could regulate gene expression by modulating the accessibility of chromatin, and epigenetic alteration of histone modification has been described as major contributors to cellular aging.^35^, ^36^ Increased histone modifications of H4K16ac, or H3K4me3, as well as decreased H3K9me3 or H3K27me3, constitute aging‐associated epigenetic marks.^37^ We reasoned ALKBH5 could be regulated in an epigenetic regulation manner by histone modification. To verify whether expression of ALKBH5 could be regulated by histone epigenetic modification, we used online tool (ENCODE) to predict above four kinds of histone modification of ALKBH5 promoter (Figure 2A), and ChIP‐qPCR was performed to identify their change levels. Results showed the H3K9me3 at the promoter changed most significantly with downregulation after treatment with TNFα (Figure 2B‐E). Data from transcriptome sequencing revealed, KMT1A and KDM4A, two transferases mediating H3K9me3 modification, significantly changed in NPCs (Figure 2F). And western blot of normal and degenerated tissues further confirmed above results, accompanied with a more significant change of KDM4A than KMT1A (Figure 2G, H). Additionally, slightly increased KMT1A functions as a methyltransferase, which would lead to the opposite direction of H3K9me3 modification. Therefore, KDM4A was chosen as the potential contributor to decrease H3K9me3 modification of ALKBH5, in accordance with the transcript sequencing data in senescent NPCs (Figure S2A). And then gain of function assays of KDM4A in NPCs further confirmed KDM4A was an effective contributor to H3K9me3 (Figure 2I, J), and DNA‐pulldown assay also revealed KDM4A bound more to the promoter region of ALKBH5 in senescent NPCs^38^ (Figure 2K, L). Further ChIP‐qPCR showed, compared with control NPCs, more KDM4A combined with ALKBH5 at the promoter region in senescent NPCs (Figure 2M). Loss of H3K9me3 correlates with the block of gene expression by inhibiting Pol II recruitment to the promoter sites. In normal NPCs, overexpression of demethylase KDM4A could down‐regulate H3K9me3 and promote Pol II recruitment and expression of ALKBH5, while inhibition of H3K9me3 demethylases using JIB‐04 in senescent NPCs leads to the opposite outcome (Figure 2N–P). Consistently, DNase I sensitivity assay also showed consistent change of chromatin accessibility of the ALKBH5 promoter in KDM4A‐overexpressed and TNFα‐induced senescent NPCs^39^ (Figure 2Q). Collectively, the above data demonstrated upregulation of ALKBH5 in the senescence process of NPCs was due to epigenetic decrease of H3K9me3 in the promoter.

### 3.3 NPC senescence is accompanied with DNMT3B m6A hypomethylation

**FIGURE 3:**
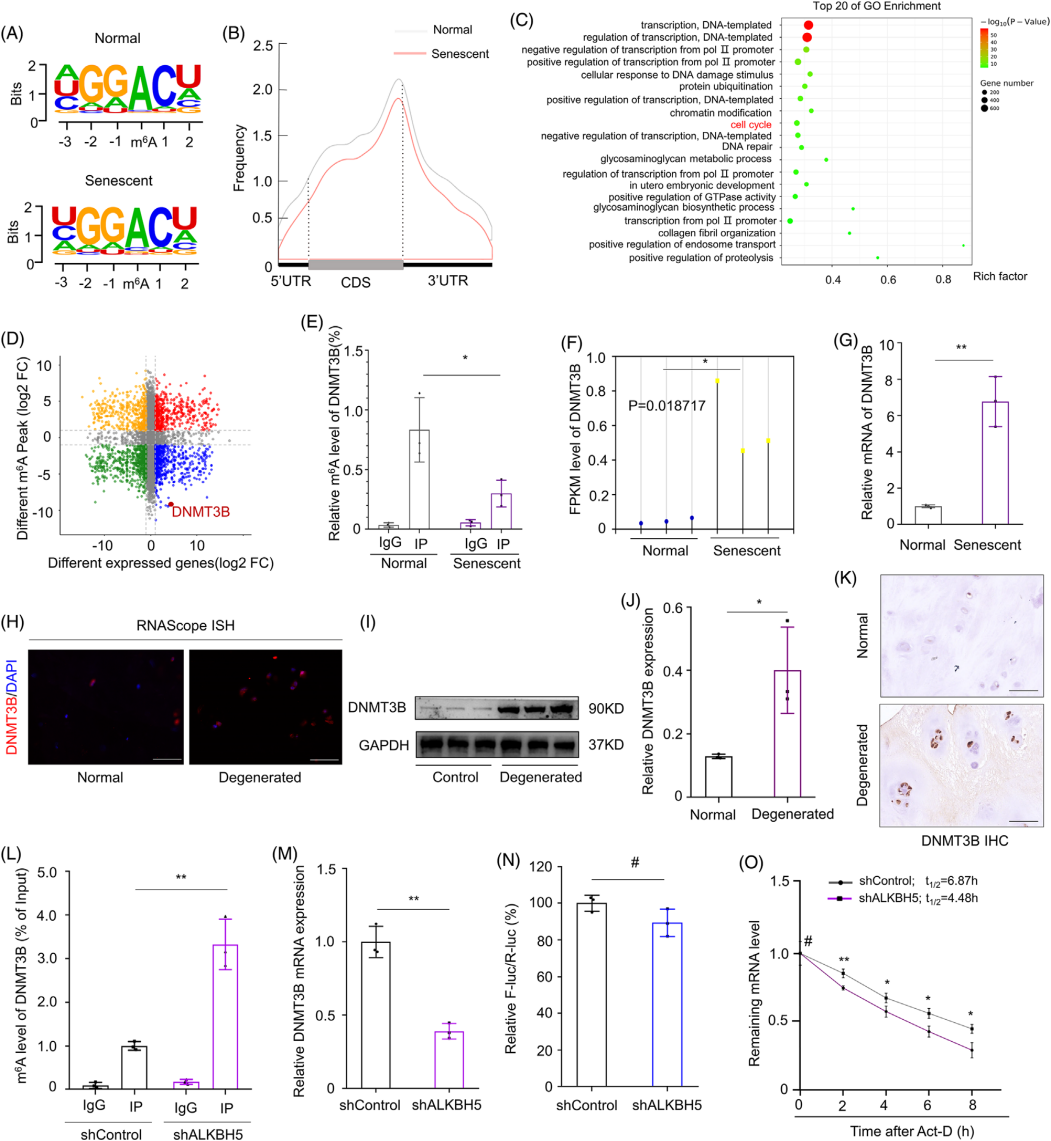
NPC senescence was accompanied with DNMT3B m6A hypomethylation. (A) Motif of m6A peak of Me‐RIP‐Seq of transcripts in normal and senescent NPCs. (B) Distribution of m6A peak in transcripts in normal and senescent NPCs, *n* = 3. (C) Top 20 related pathways and enriched cellular processes of gene ontology (GO) enrichment analysis of genes harbouring different m6A peaks. (D) Integrating analysis data of different genes from m6A‐seq and corresponding RNA‐seq data. (E) m6A modification of DNMT3B transcripts by Me‐RIP‐qPCR during cellular senescence, IgG was used as a negative control, *n* = 3. *P < 0.05, two‐tailed unpaired Student's *t*‐test. (F) Analysis of DNMT3B FPKM in the transcript sequencing data of normal and senescent NPCs, *n* = 3. *P < 0.05, two‐tailed unpaired Student's *t*‐test. (G) mRNA level analysis of DNMT3B in normal and senescent NPCs by RT‐qPCR, GAPDH was used as a loading control, *n* = 3. **P < 0.01, two‐tailed unpaired Student's *t*‐test. (H) mRNA expression of DNMT3B in normal and degenerated NPC tissues by RNA Scope ISH (scale bar: 50 μm). (I, J) Protein level analysis of DNMT3B in normal and degenerated NPC tissues by western blot, GAPDH was used as a loading control, *n* = 3. *P < 0.05, two‐tailed unpaired Student's *t*‐test. (K) IHC analysis of DNMT3B expression in normal and degenerated NP tissues (scale bar: 50 μm). (L) m6A modification of DNMT3B transcripts by Me‐RIP‐qPCR in ALKBH5‐silenced NPCs, IgG was used as a negative control, *n* = 3. **P < 0.01, two‐tailed unpaired Student's *t*‐test. (M) mRNA level analysis of DNMT3B in NPCs with or without ALKBH5 silencing by RT‐qPCR, GAPDH was used as a loading control, *n* = 3. **P < 0.01, two‐tailed unpaired Student's *t*‐test. (N) Promoter activity analysis of DNMT3B using dual luciferase reporter assay in wild‐type and ALKBH5‐knockdown NPCs, *n* = 3. ^#^ *P *> 0.05, two‐tailed unpaired Student's *t*‐test. (O) Stability of mature mRNA of DNMT3B in ALKBH5‐knockdown or control NPCs, *n* = 3. ^#^ *P *> 0.05, *P < 0.05, **P < 0.01, two‐tailed unpaired Student's *t*‐test

RNA methylation could affect the turnover of mRNA transcripts via regulating the methylation level of target transcripts. To characterise the potential targets involved in m6A‐regulated senescence of NPCs, we performed Me‐RIP‐seq (m6A‐seq) to map the m6A methylomes of NPCs undergoing senescence with three independent biological replicates. And data manifested m6A sites were highly enriched in consensus GGAC motif in both senescent and control NPCs, especially abundant in the vicinity of start and stop codons (Figure 3A, B). To characterize the functional role of m6A modification, we identified the related pathways and enriched cellular processes of genes harbouring different m6A peaks, and “cell cycle” was demonstrated among the top significant biological processes (Figure 3C). By integrating analysis data of m6A‐seq and corresponding RNA‐seq data, we identified that DNMT3B was modified with m6A and one of the top genes with significant fold change of m6A modification during cellular senescence, which was confirmed by Me‐RIP‐qPCR analysis (Figure 3D, E). Moreover, consistent with RNA‐seq data, the mRNA expression of DNMT3B increased significantly during NPC senescence (Figure 3F, G). Furthermore, expression variance of DNMT3B transcripts in normal and degenerated NP tissues was confirmed by RNAScope ISH (Figure 3H and Figure S3A). Western blot was performed to find the protein level that was increased as well, in line with immunochemistry results of NP tissues from degenerated discs (Figure 3J, K and Figure S3B).

To further illuminate the role of m6A modification on DNMT3B expression, we examined the methylation level of DNMT3B transcripts by Me‐RIP‐qPCR in ALKBH5 knockdown NPCs and found that the m6A modification of DNMT3B was obviously increased under inhibition of ALKBH5, in concordance with the change of global m6A modification (Figure 3L and Figure S3C). Meanwhile, the expression of DNMT3B at both mRNA and protein level declined accompanied with ALKBH5 knockout‐induced higher methylation (Figure 3M and Figure S3E), indicating DNMT3B was regulated by m6A modification during NPC senescence. Therefore, the mechanisms regulating DNMT3B expression through m6A methylation demand further investigation.

First, we tested the promoter activity of DNMT3B using dual luciferase reporter assay in wild type and ALKBH5 knockdown NPCs and found that there was no obvious difference, suggesting that the transcription process of DNMT3B was not influenced by m6A (Figure 3N). Expression of pre‐mRNA (precursor) detected by RT‐qPCR showed no difference was observed in pre‐mRNA expression (Figure S3D). Then, we separated RNAs in nuclear and cytoplasm, and observed no different subcellular localization of DNMT3B mRNAs, which explains the fact that m6A could not affect the exportation of transcripts (Figure S3F, G). Furthermore, NPCs were treated with Cycloheximide (CHX) to block translation and western blot results verified that half‐life of DNMT3B proteins were similar, suggesting that m6A‐associated DNMT3B expression was not related to protein stability (Figure S3H). Moreover, ribosome–nascent chain complex qPCR (RNC–qPCR) was used to assess the translational efficiency of DNMT3B, whereas no difference of polysomes bound to EGFR mRNA in wild type and ALKBH5‐silencing NPCs showed a negative result, revealing translation efficiency of DNMT3B in ALKBH5‐silencing NPCs was consistent with that in wild type cells^40^ (Figure S3I). Above data indicated that there is no significant relationship between m6A modification and DNMT3B translation. Next, we treated NPCs with Act‐D to block the transcription, and the half‐life of pre‐RNA (precursor) and mat‐RNA (mature) was detected by RT‐qPCR at multi‐point time. The stability of mature mRNA of DNMT3B in ALKBH5‐knockdown NPCs was declined with shorter half‐life (Figure 3O). These results demonstrated that m6A modification may affect the degradation of mature mRNA but not the splicing of precursor mRNA of DNMT3B during NPC senescence.

### 3.4 m6A‐methylated 3′UTR regulates decay of DNMT3B mRNA

**FIGURE 4:**
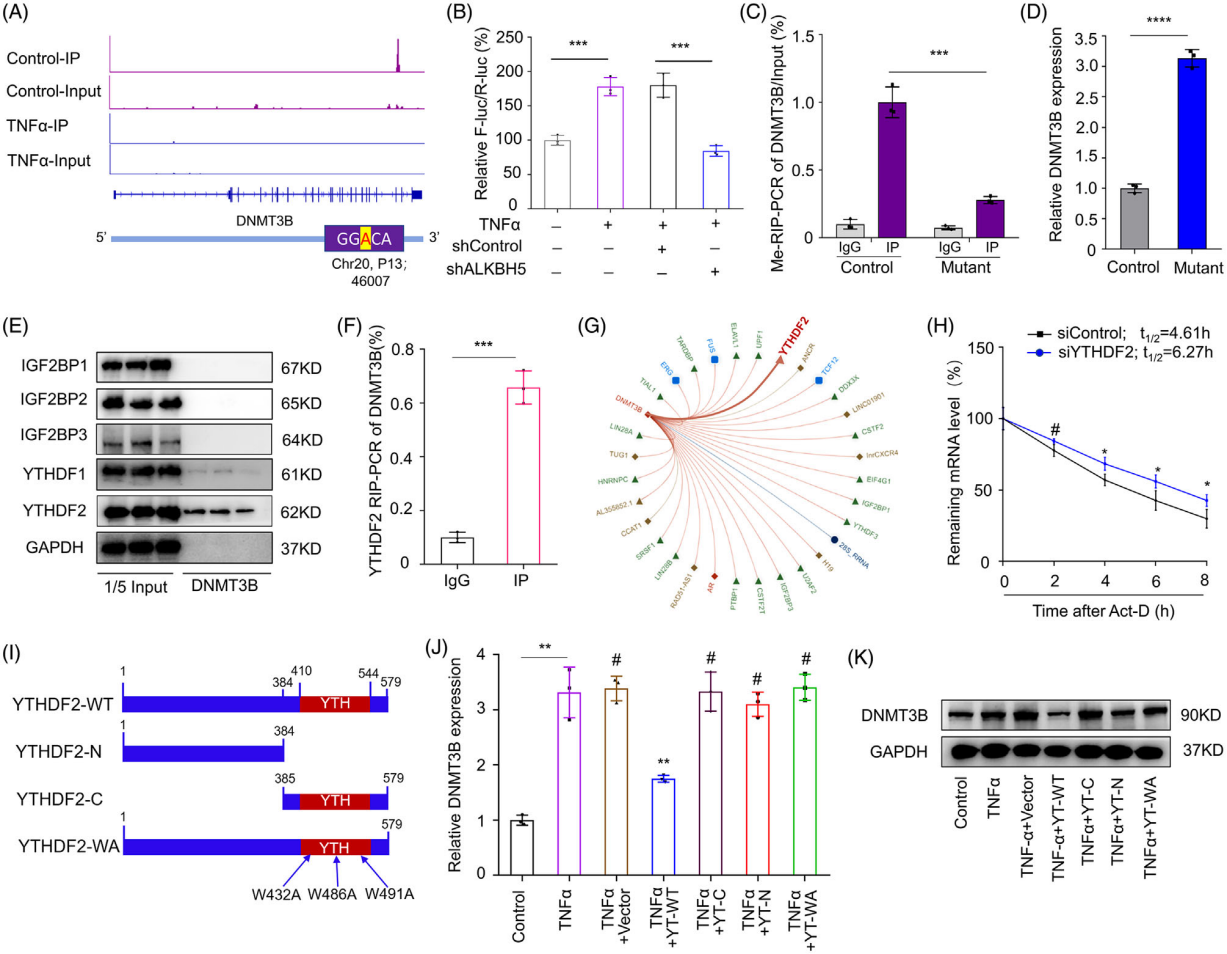
m6A‐methylated 3′UTR regulates decay of DNMT3B mRNA. (A) Analysis of sequencing data combined with bioinformatic analysis of the IP and input data of control and TNFα groups showing one GGACU motif in the 3′UTR region of DNMT3B mRNA, the vertical axis represents signal intensity. (B) Dual luciferase reporter assay of luciferase activity with or without ALKBH5 knockdown. (C, D) m6A modification level of DNMT3B by Me‐RIP‐qPCR and expression by RT‐qPCR in NPCs with or without point mutation, *n* = 3. ***P < 0.001, ****P < 0.0001, two‐tailed unpaired Student's *t*‐test. (E) RNA‐pull down followed by western blot analysis of the isolated proteins of stability‐associated readers. (F) Confirmation the recognition of YTHDF2 with DNMT3B mRNA by RIP‐qPCR. (G) Prediction of recognition by YTHDF2 of DNMT3B mRNA using the online tool database *RNA Intern*. (H) mRNA stability assay of DNMT3B in NPCs with or without YTHDF2 silencing, *n* = 3. ^#^ *P *> 0.05, *P < 0.05, two‐tailed unpaired Student's *t*‐test. (I–K) Expression of DNMT3B in NPCs with or without introduction of YTHDF2 truncation mutants by RT‐qPCR and western blot analysis, GAPDH was used as a loading control, *n* = 3. ^#^ *P *> 0.05, **P < 0.01, two‐tailed unpaired Student's *t* test

To further clarify the regulatory mechanism of m6A on DNMT3B transcripts, we analysed the Me‐RIP‐seq data which indicated the m6A peak of DNMT3B was enriched in 3′UTR regions. Analysis of the sequencing data combined with bioinformatic analysis showed one GGACU motif in the 3′UTR region of DNMT3B mRNA (Figure 4A), consistent with the position of peaks identified by Me‐RIP‐seq. Furthermore, we generated luciferase reporters containing a firefly luciferase placed before the wild type and mutant DNMT3B‐3′UTR with substitution of A of the m6A sites with G. The dual luciferase reporter assay revealed that TNFα treatment could downregulate the luciferase activity, which could be partially abrogated by knockdown of ALKBH5 (Figure 4B), and Me‐RIP‐qPCR showed mutation could decrease the m6A modification of DNMT3B (Figure 4C, D), further confirming the methylation site of DNMT3B.

Previous study verified “readers” that could influence the stability of m6A‐modified transcripts include YTHDF2/3 and IGF2BP1/2/3.^41^ To identify the molecular mechanism by which m6A modification regulates the stability of DNMT3B mRNA, RNA‐pull down was performed followed by western blot analysis of the isolated proteins in senescent NPCs. Results manifested YTHDF2 interacted stronger with DNMT3B mRNA, and additional RIP‐qPCR analysis further revealed above recognition (Figure 4E, F), consistent with the prediction result of RNA interaction database *RNA Intern* (Figure 4G). In both normal and senescent NPCs, inhibition of YTHDF2 could lead to higher stability and expression of DNMT3B mRNA (Figure 4H). We then introduced the YTHDF2 truncation mutants (YTHDF2‐N and YTHDF2‐C) and m6A recognition site mutant (YTHDF2‐WA) in the NPCs (Figure 4I), and results at protein and transcript level implied that overexpression of full‐length but not mutants could promote the mRNA decay of DNMT3B (Figure 4J, K). These results demonstrated that the effect of m6A on DNMT3B was triggered by YTHDF2‐mediated decay of transcripts.

### 3.5 DNMT3B promotes senescence of NPCs during IVD degeneration

**FIGURE 5:**
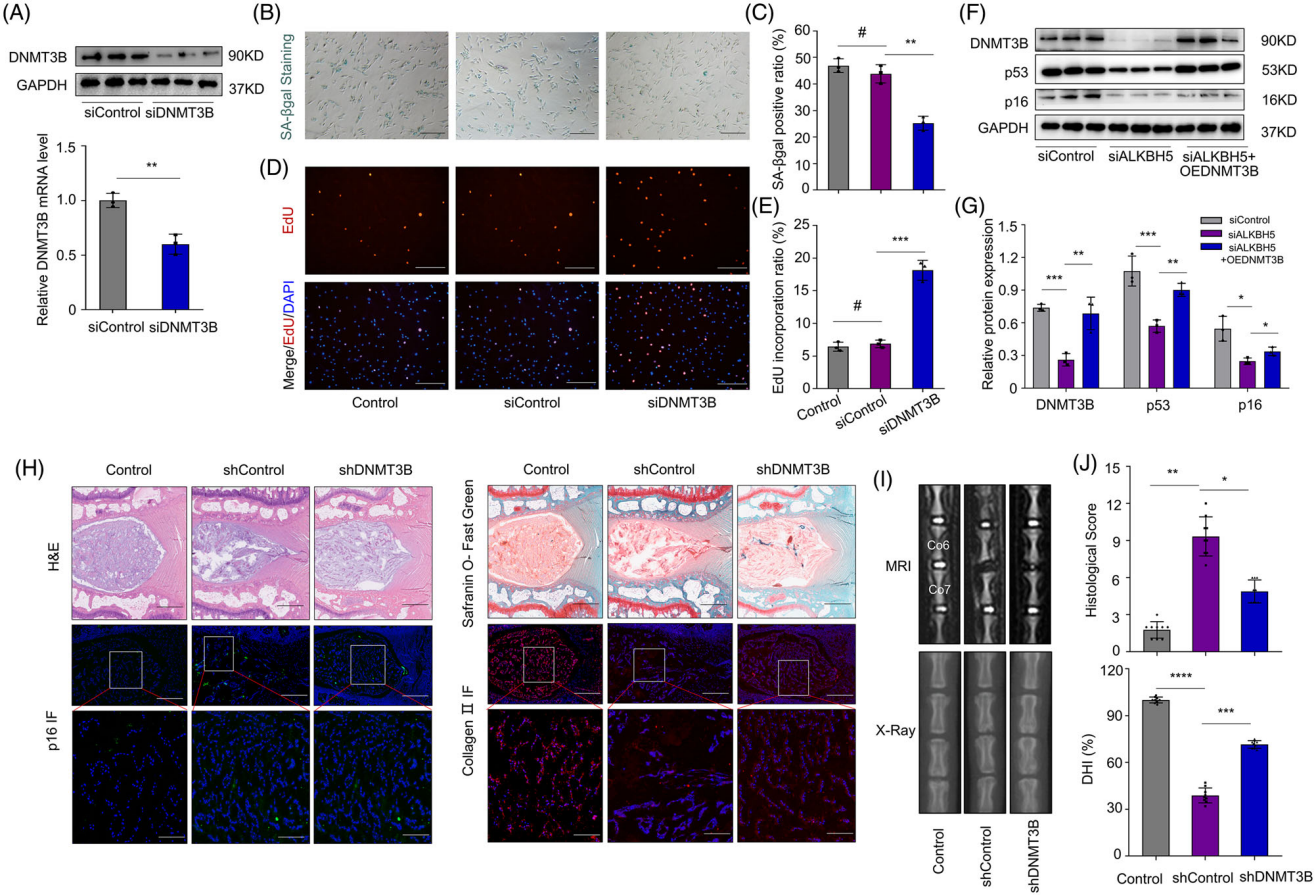
DNMT3B promotes senescence of nucleus pulposus cell (NPCs) in intervertebral disc (IVD) degeneration. (A) Knockdown efficiency of DNMT3B in NP cells by siRNA, analysed by western blot and RT‐qPCR, GAPDH was used as a loading control, *n* = 3. **P < 0.01, two‐tailed unpaired Student's *t*‐test. (B, C) SA‐β‐gal activity staining (scale bar: 50 μm) and analysis of NPCs with or without DNMT3B silencing, *n* = 3. ^#^ *P *> 0.05, **P < 0.01, two‐tailed unpaired Student's *t*‐test. (D, E) EdU incorporation assay (scale bar: 100 μm) of NPCs with or without DNMT3B silencing, *n* = 3. ^#^ *P *> 0.05, ***P < 0.001, two‐tailed unpaired Student's *t*‐test. (F, G) Expression of senescent markers in NPCs with or without DNMT3B silencing, *n* = 3. *P < 0.05, **P < 0.01, ***P < 0.001, two‐tailed unpaired Student's *t*‐test. (H) Histological staining by H&E, Safranin O‐Fast Green, and IF of a rat model of IVD degeneration (scale bar: 500 μm; 100 μm) with or without DNMT3B silencing; Control represents group without surgery, shControl represents group with surgery using lentivirus containing shControl, shDNMT3B represents group with surgery using lentivirus containing shDNMT3B. (I) Radiographic imaging of a rat model of IVD degeneration with or without DNMT3B silencing by MRI and X‐ray. (J) Analysis of histological staining, *n* = 9. *P < 0.05, **P < 0.01, ***P < 0.001, ****P < 0.0001, two‐tailed unpaired Student's *t*‐test

To further characterize the function of DNMT3B in the NPC senescence and disc degeneration, we knocked down the expression of DNMT3B with siRNA in NPCs, and knock down‐efficiency was verified by western blot and RT‐qPCR (Figure 5A). Compared with wild‐type NPCs treated with TNFα, the senescence of NPCs with deficiency of DNMT3B expression was alleviated. NPCs with DNMT3B silencing showed decreased SA‐β‐gal activity (Figure 5B, C), consistent with EdU incorporation analysis, indicating increased ratio of EdU positive cells and stronger ability of self‐renewal (Figure 5D, E). Furthermore, overexpression of DNMT3B could abrogate the anti‐senescence effect of ALKBH5 knockdown in NPCs, manifested by relative up‐regulation of senescence markers (Figure 5F, G). In the rat model of IVD degeneration, we revealed silencing of DNMT3B using lentivirus containing shRNA could alleviate the degeneration of IVD and the aging process of NPCs in vivo, reflected by MRI imaging and histological analysis (Figure 5H‐J and Figure S4A).

### 3.6 DNMT3B elevation causes E4F1 promoter hypermethylation and E4F1 suppression to promote IVD degeneration

**FIGURE 6:**
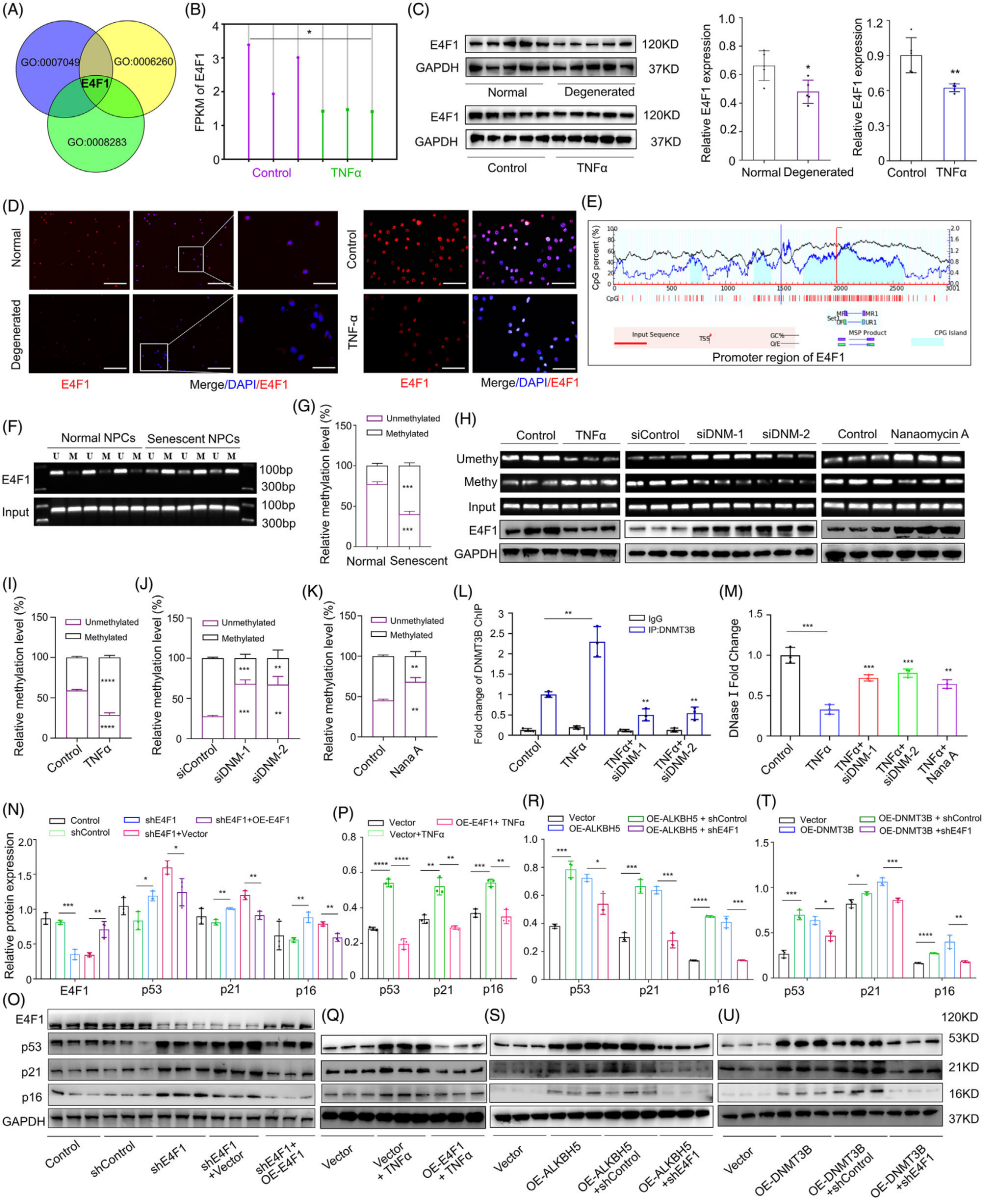
DNMT3B elevation causes E4F1 promoter hypermethylation and E4F1 suppression to promote intervertebral disc (IVD) degeneration. (A) Bioinformatic analysis of genes associated with cell cycle and senescence. (B) Analysis of DNMT3B FPKM in the transcript sequencing data of normal and senescent NPCs, *n* = 3. *P < 0.05, two‐tailed unpaired Student's *t*‐test. (C) Expression analysis of E4F1 in normal or degenerated NP tissues or in normal and senescent NPCs, *n* = 3. *P < 0.05, **P < 0.01, two‐tailed unpaired Student's *t*‐test. (D) IF analysis of E4F1 expression in normal and degenerated NP tissues or in normal and senescent NPCs (red: E4F1; blue: DAPI; scale bar: 100 μm; 50 μm). (E) Schematic diagram and CpG ratio of E4F1 promoters (−2000/+1000) by *Meth Primer* software (https://www.urogene.org/methprimer/). (F, G) Methylation‐specific PCR (MSP) and analysis of the methylation level of E4F1 in NPCs from normal and degenerated NP tissues, *n* = 3. ***P < 0.001, two‐tailed unpaired Student's *t*‐test. (H–K) Methylation level of E4F1 by MSP and expression by western blot in normal and senescent NPCs, DNMT3B‐silenced NPCs and NPCs treated with the DNMT3B inhibitor Nanaomycin A, *n* = 3. **P < 0.01, ***P < 0.001, two‐tailed unpaired Student's *t*‐test. (L) ChIP‐qPCR analysis of DNMT3B recruitment to the E4F1 promoter region in normal or senescent NPCs with or without DNMT3B silencing, IgG was used as a negative control, *n* = 3. **P < 0.01, two‐tailed unpaired Student's *t*‐test. (M) Chromatin accessibility of the E4F1 promoter in normal or senescent NPCs with DNMT3B silencing or Nanaomycin A treatment analysed by DNase I sensitivity assay, *n* = 3. **P < 0.01, ***P < 0.001, two‐tailed unpaired Student's *t*‐test. (N, O) Protein expression of E4F1 and senescence indicators in control or E4F1‐silenced NPCs with E4F1 overexpression or not, *n* = 3. *P < 0.05, **P < 0.01, ***P < 0.001, two‐tailed unpaired Student's *t*‐test. (P, Q) Protein expression of senescence indicators in senescent NPCs with or without E4F1 overexpression, *n* = 3. **P < 0.01, ***P < 0.001, ****P < 0.0001, two‐tailed unpaired Student's *t*‐test. (R, S) Protein expression of senescence indicators in ALKBH5‐overexpressing NPCs with or without E4F1 silencing, *n* = 3. *P < 0.05, ***P < 0.001, two‐tailed unpaired Student's *t*‐test. (T, U) Protein expression of senescence indicators in DNMT3B‐overexpressing NPCs with or without E4F1 silencing, *n* = 3. *P < 0.05, **P < 0.01, ***P < 0.001, ****P < 0.0001, two‐tailed unpaired Student's *t*‐test

DNMT3B is a DNA methyltransferase that could suppress the expression of target genes via hypermethylation of the CpG islands in the promoter regions. Analysing the sequencing data combined with bioinformatic analysis of genes associated with cell cycle and senescence (GO:0006260: DNA replication; GO:0008283: cell proliferation; GO:0007049: cell cycle), we found that E4F1 had a significant difference (Figure 6A, B). Furthermore, the variant expression of E4F1 in senescent NPCs was confirmed by western blot and immunofluorescence, consistent with the results analysed in NP tissues (Figure 6C, D). To further investigate whether the E4F1 could be regulated by DNA methylation, we examined the methylation status of E4F1 promoter (−2000/+1000) in NPCs by Methylation‐specific PCR (MSP) analysis according to *MethPrimer* software recommendations (Figure 6E). The results showed, compared with normal NPCs, that senescent NPCs from degenerated individuals exhibited increased level of methylation at E4F1 promoter (Figure 6F, G). Furthermore, human NPCs treated with TNFα also manifested the same methylation tendency in E4F1 promoter, whereas additional administration of Nanaomycin A, a specific inhibitor of DNMT3B, and siRNAs against DNMT3B in NPCs could abolish the methylation level to some extent, and increase the expression of E4F1 meanwhile (Figure 6H–K). To gain additional molecular mechanism insight into the role of DNMT3B in the regulation of E4F1 gene, ChIP‐qPCR was performed to investigate the recruitment of DNMT3B to the promoter region of E4F1. Increased occupancy of DNMT3B in the CpG region of E4F1 was observed in senescent NPCs by ChIP assay, which could be abolished by DNMT3B silencing (Figure 6L). Furthermore, a notable decrease of chromatin accessibility of the E4F1 promoter in senescent NPCs was observed by DNase I sensitivity assay, which could be alleviated by inhibitor or small interference RNA of DNMT3B (Figure 6M). Collectively, these results demonstrate that DNMT3B elevation causes E4F1 promoter hypermethylation and suppresses E4F1 expression in NPC senescence.

E4F1 is a key regulator involved in proliferation and survival of cells, and is an essential gene in embryonic stem cells and during early embryogenesis.^42^, ^43^, ^44^ However, the roles of E4F1 in disc degeneration and NPC senescence remain unclear. Therefore, we investigated whether E4F1 repression devoted to the senescence of NPCs using lentivirus containing shRNA. As shown in Figure 6N–Q, downregulation of E4F1 led to autogenous senescence of NPCs, while overexpression of E4F1 in NPCs could reverse the senescence induced by TNFα. Furthermore, enforced expression of E4F1 in NPCs could abolish the pro‐senescence effect of ALKBH5 and DNMT3B to some extent (Figure 6R–U), demonstrating deficiency of E4F1 contributes to senescence of NPCs and revealing the critical role of m6A/DNMT3B/E4F1 axis in NPC senescence.

## 4 DISCUSSION

Among over 100 types of RNA modifications, m6A is the most prevalent one in modification of mRNA and non‐coding RNA.^45^ Accumulating evidences indicate that m6A is involved in a series of physical and pathological processes, including embryonic stem cells development, tumorigenesis, spermatogonia differentiation, CNS myelination, heart failure and diabetes.^46^, ^47^, ^48^, ^49^, ^50^, ^51^, ^52^, ^53^, ^54^ Recent studies revealed that demethylase FTO could control cell cycle progression and proliferation by targeting m6A modification of cyclin D1 mRNA.^32^ What's more, expression of miRNA in cellular aging of HDFs and old PBMCs could be regulated by m6A‐dependent AGO2 expression.^55^ With the progression of aging, NPCs undergo senescence, which contributes to degeneration of IVD. In the present study, we revealed expression of demethylase ALKBH5 was increased in aged NPCs. And in vitro experiments demonstrated that upregulation of ALKBH5 was due to epigenetic decrease of H3K9me3 modification. Deletion of ALKBH5 could suppress the in vitro senescence of NPCs and ameliorate the aging and degeneration of IVD in vivo. Me‐RIP‐seq and analysis were performed and results showed that DNMT3B was dynamically methylated at 3′UTR regions and significantly regulated by m6A during NPC senescence. Mechanistically, up‐regulation of ALKBH5 suppressed m6A modification of DNMT3B transcripts, resulting in less recognition of YTHDF2 and thus inhibiting the decay effect. Stabilized mRNA promoted the expression of DNMT3B and led to the promoter hypermethylation of E4F1, inhibiting the expression of E4F1, which contributes a lot to the senescence of NPCs.

The roles of m6A and ALKBH5 in many diseases and pathological processes have been investigated extensively. In breast cancer, hypoxia induces expression of ALKBH5 in an Hif‐dependent manner, and increased ALKBH5 causes decreased m6A modification of NANOG mRNA and increased expression due to higher stability, increasing the percentage of breast cancer stem cells and inducing their capacity for tumour initiation.^56^ ALKBH5 could target transcription factor FOXM1 and promote the tumour proliferation of glioblastoma stem‐like cells, which could be enhanced by a nuclear lncRNA FOXM1‐AS.^57^ In the virus–host interaction, host cells actively respond to viral infection by impairing m6A demethylation activity of ALKBH5 through demethylating R107 residue in protein, leading to the increased mRNA degeneration and inhibited protein expression of Oxoglutarate Dehydrogenase (OGDH), limiting the TCA cycle and cellular metabolism in host cells, thus restricting viral infection.^58^ Furthermore, recent investigation by Wang et al. in AML development revealed ALKBH5, affected by chromatin accessibility through KDM4C, is required in the maintenance of the leukaemia stem cell function by affecting the stability of AXL receptor tyrosine kinase (AXL) transcripts in an m6A‐dependent manner.^38^ Here, we first depicted that key DNA methylation regulator DNMT3B could be regulated by ALKBH5 in an m6A‐modification manner, thus influencing the expression of downstream genes by methylating the CpG islands in promoter region, revealing a new landscape of epigenetic modification and regulation in NPC senescence. However, whether other transcripts with change of m6A modification have function in the aging of NPCs is worthy of more investigations.

m6A modification modulates various cellular processes via different mechanisms. After modification with m6A by balancing function of “readers” and “erasers”, transcripts with m6A will undergo different fate through RNA structure alternation or specific “readers” recognition. m6A modification on precursor mRNAs could change the local structure and accessibility of flanking RNA to recruit splicing factors such as hnRNPC and hnRNPG, affecting mRNA splicing process. Selective recognition of m6A sites in mRNA by YTHDF1 could promote the interaction with initiation factor, translation initiation and protein synthesis.^59^ However, YTHDF2 accelerates the decay of m6A‐modified mRNAs by bringing them to mRNA decay sites and destabilises transcripts by triggering deadenylation and degradation.^60^ On the contrary, IGF2BP family could interact with HuR, MATR3, and PABPC1 to protect m6A‐modified mRNAs from degradation and facilitate mRNA translation as well.^27^ In this study, our luciferase and mutation assays revealed that mRNAs of DNMT3B in senescent NPCs were accompanied with less m6A modification in 3′UTR, which abrogates the pro‐decay effect of YTHDF2, thus to promote its expression.

More and more researches revealed epigenetic regulation mechanism of NPC cellular dysfunction during IVD degeneration.^61^, ^62^ Our data demonstrated DNMT3B and the DNA methylation of E4F1 promoters functioned vitally in the senescence process of NPCs. The roles and function of DNMT3B in NPC senescence and disc degeneration were further ascertained by gain‐ and loss‐of‐function experiments in NPCs. E4F1 is a traditional transcription factor that controls genes involved in cell proliferation and cell cycle arrest, affecting the division and survival of cells.^63^ Furthermore, E4F1 affects the process of DNA damage response by regulating CHK1, a DNA damage responsive checkpoint protein, which participates in the DNA repair process under DNA damage response caused by ionizing irradiation, aging and ROS stimulation. To protect the integrity of the genome, CHK1 gets activated by phosphorylation by ATR following DNA damage to arrest the cell cycle and ensure that cells do not enter mitosis,^64^ and impaired DNA repair is sufficient to cause accelerated aging phenotypes.^65^ Moreover, another study indicated that E4F1 is involved in PDH‐dependent metabolic program to maintain epidermal stem cell and skin homeostasis.^66^ In the present study, we clarified the critical role of E4F1 in NP cellular senescence, which contributes to the degeneration of IVD.^67^ However, as for the specific regulation mechanism of E4F1, accumulation of DNA damage in E4F1‐deficiency condition may account for the senescence of NPCs.^68^ Whether there remain other regulation processes of E4F1 during NPC senescence needs further investigation.

In this study, we isolated the NPCs from normal and degenerative human discs, and although age difference between the two groups existed, the young and aged statuses of NPCs from the two groups might remain unaffected. Furthermore, a specific grading criterion for adolescents with developing lumbar spine will be superior to the Pffirmann grading system to evaluate the disc status of scoliosis lumbar IVDs and help to produce more convincing data. We clarified ALKBH5 could be regulated by H3K9me3, and as for whether other kind of histone modification manner could regulate the expression of ALKBH5, such as H3K27me3, remains unclear and further investigation is needed. Although a previous study has reported the role of DNMT3B in IVD degeneration via regulating extracellular matrix degradation,^69^ whether DNMT3B regulates NPC cellular senescence remains unknown. Besides, we knockdown or overexpression of ALKBH5 or DNMT3B or KDM4A in these rescue experiments in this study and maybe using dCase9 or dCase13b specific to the targets in the rescue experiments will provide more convincing data. Moreover, we identified the critical role of KDM4A/ALKBH5/DNMT3B/E4F1 axis in NPC cellular senescence and IVD degeneration in this study, additional targets and mechanisms may also exist and more investigation will pave a broader regulatory landscape of IVD degeneration.

**FIGURE 7:**
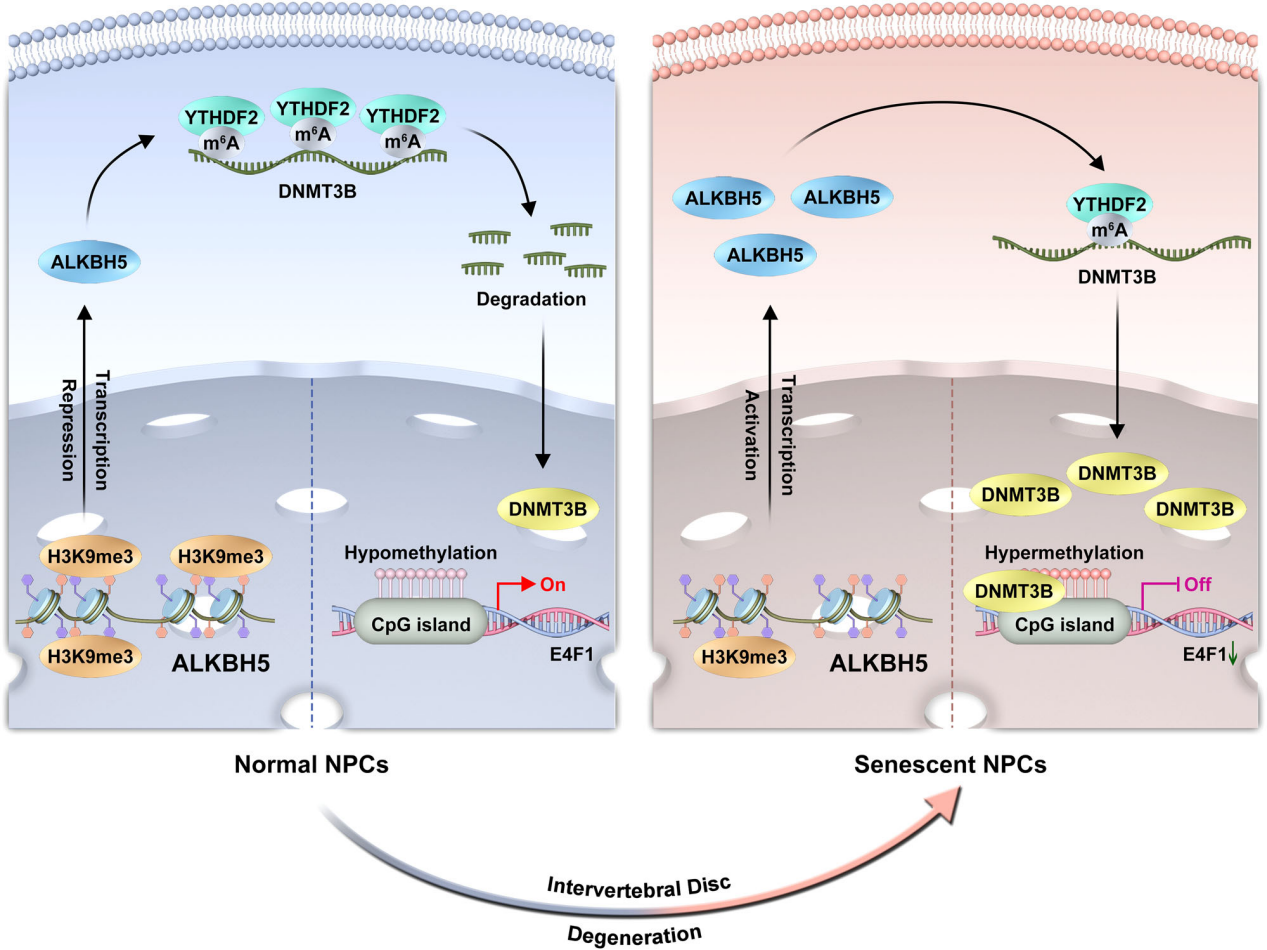
Schematic representation of mechanisms by which m6A modification mediates nucleus pulposus cell (NPC) senescence and intervertebral disc (IVD) degeneration

In summary, our data identify DNMT3B mRNA as a novel m6A modification target in NPC cellular senescence. m6A hypomethylation of DNMT3B leads to less recognition of YTHDF2, resulting in enhanced stability of DNMT3B mRNA. Increased expression of DNMT3B modulated the E4F1 level upon DNA methylation patterns causing cellular arrest and senescent phenotype (Figure 7). Here, we present that the post‐transcriptional mechanism of m6A regulated by H3K9me3 is involved with genome epigenetic modification, highlighting the crosstalk of methylation regulation from different level, including histone modification, m6A and DNA methylation during cellular senescence in our study. Therefore, ALKBH5 and the following effectors might be potential therapeutic targets for IVD degeneration.

## CONFLICT OF INTEREST

All the authors declare no conflict of interests.

## Supporting information

Supporting information

Supporting information
