# Corrigendum to “m6A hypomethylation of DNMT3B regulated by ALKBH5 promotes intervertebral disc degeneration via E4F1 deficiency”

**DOI:** 10.1002/ctm2.1353

**Published:** 2023-08-01

Li G, Luo R, Zhang W, et al. m6A hypomethylation of DNMT3B regulated by ALKBH5 promotes intervertebral disc degeneration via E4F1 deficiency. Clinical and Translational Medicine. 2022;12.

In this article, the EdU images in Figure 5D of the Control group and siControl group were misused identically. The image in lower panel of GAPDH in Figure 5F was misused. The images in lower panel of p16 IF staining of shcontrol and shDNMT3B groups in Figure 5H were misused identically. The updated Figure 5 is provided.

**FIGURE 5:**
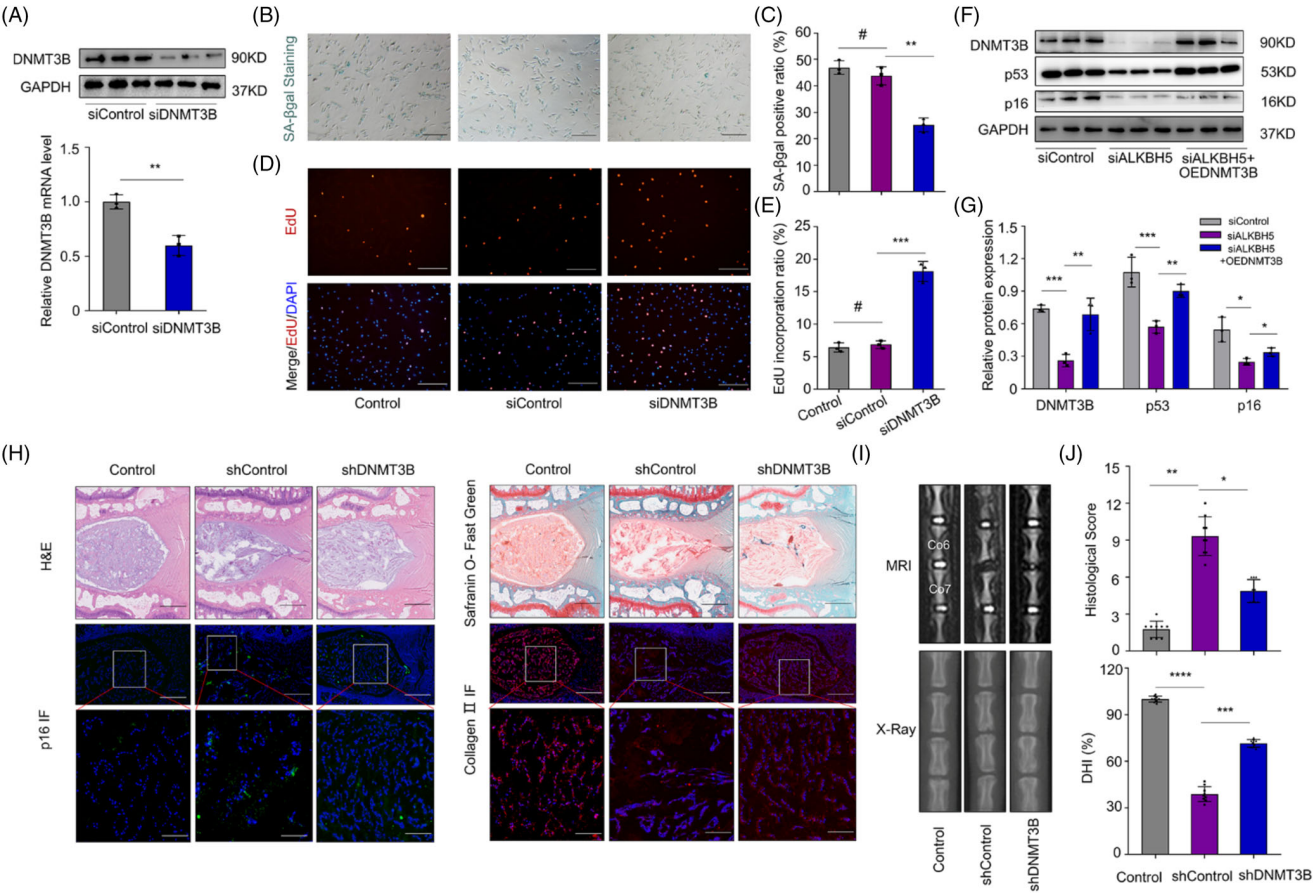


In this article, the image in lower panel of GAPDH in Figure 3I was misused. The updated Figure 3 is provided.

**FIGURE 3:**
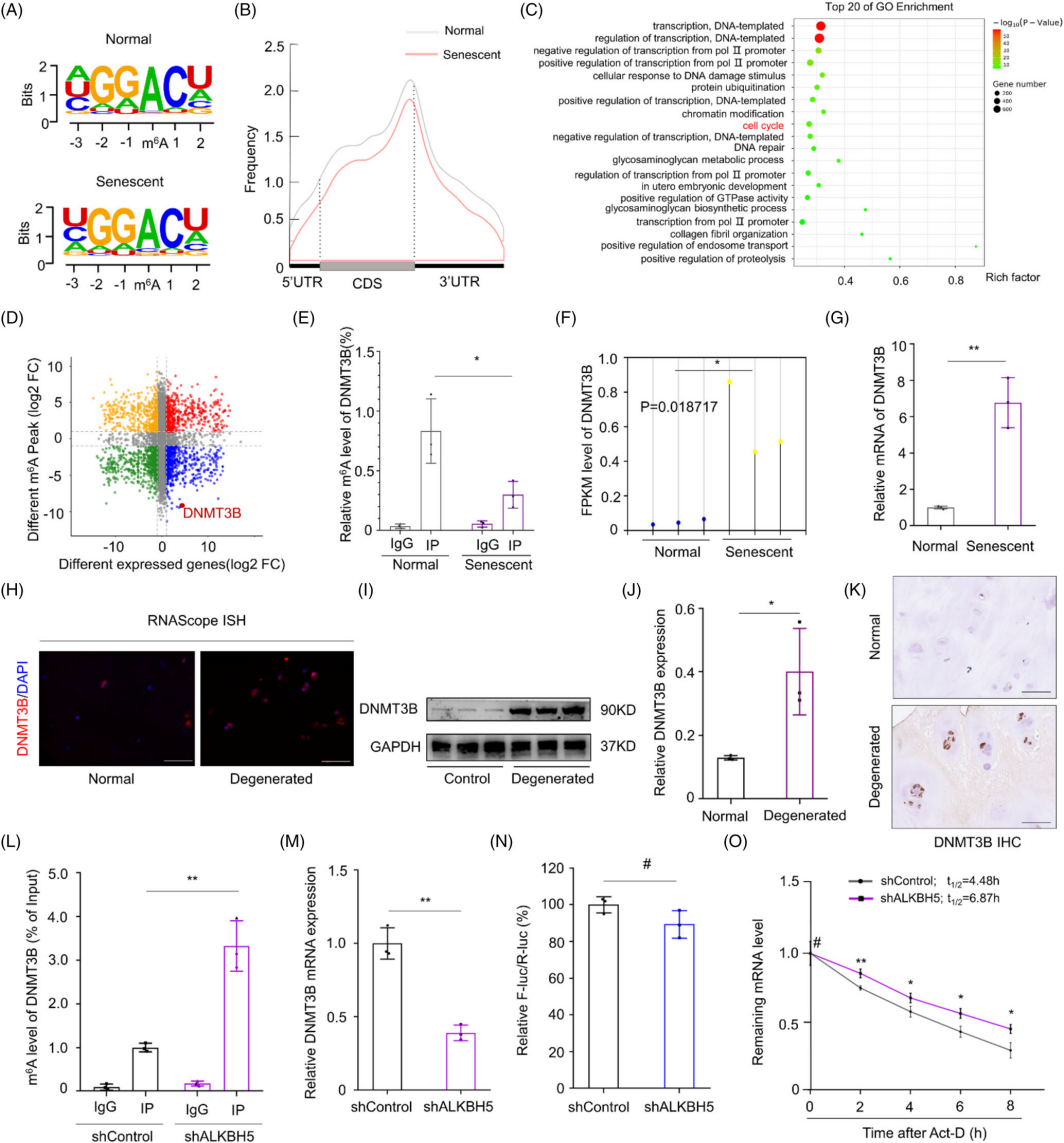


We apologize for this error.

