# Corrigendum to “m6A hypomethylation of DNMT3B regulated by ALKBH5 promotes intervertebral disc degeneration via E4F1 deficiency”

**DOI:** 10.1002/ctm2.70528

**Published:** 2025-11-20

Cao Yang

Department of Orthopaedics, Union Hospital, Tongji Medical College, Huazhong University of Science and Technology

Li G, Luo R, Zhang W, et al. m6A hypomethylation of DNMT3B regulated by ALKBH5 promotes intervertebral disc degeneration via E4F1 deficiency. *Clinical and Translational Medicine*. 2022;12.

In this article, the image in the upper panel of ALKBH5 in Figure 1I was misused. The updated Figure 1 is provided.


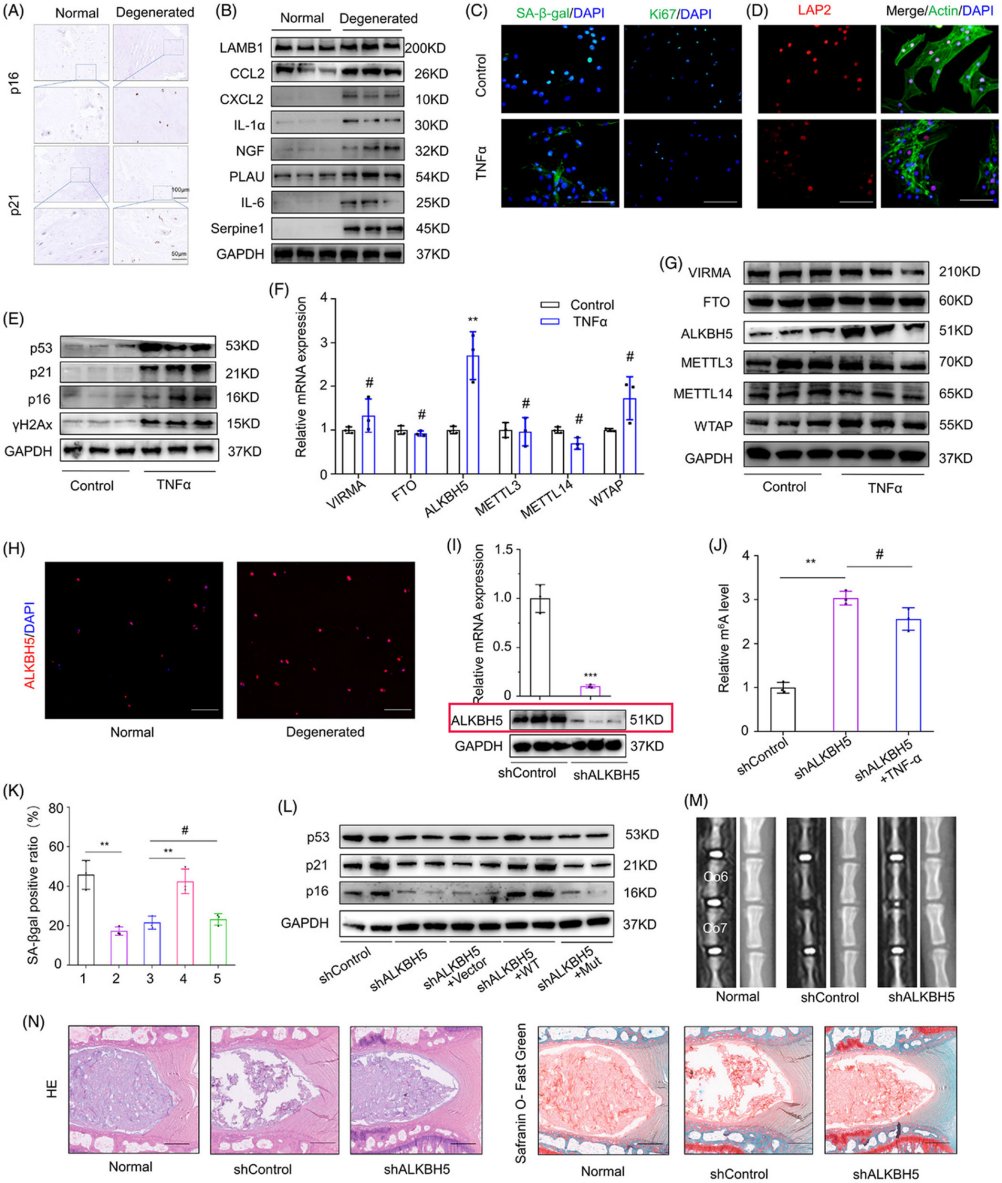


FIGURE 1 Original version


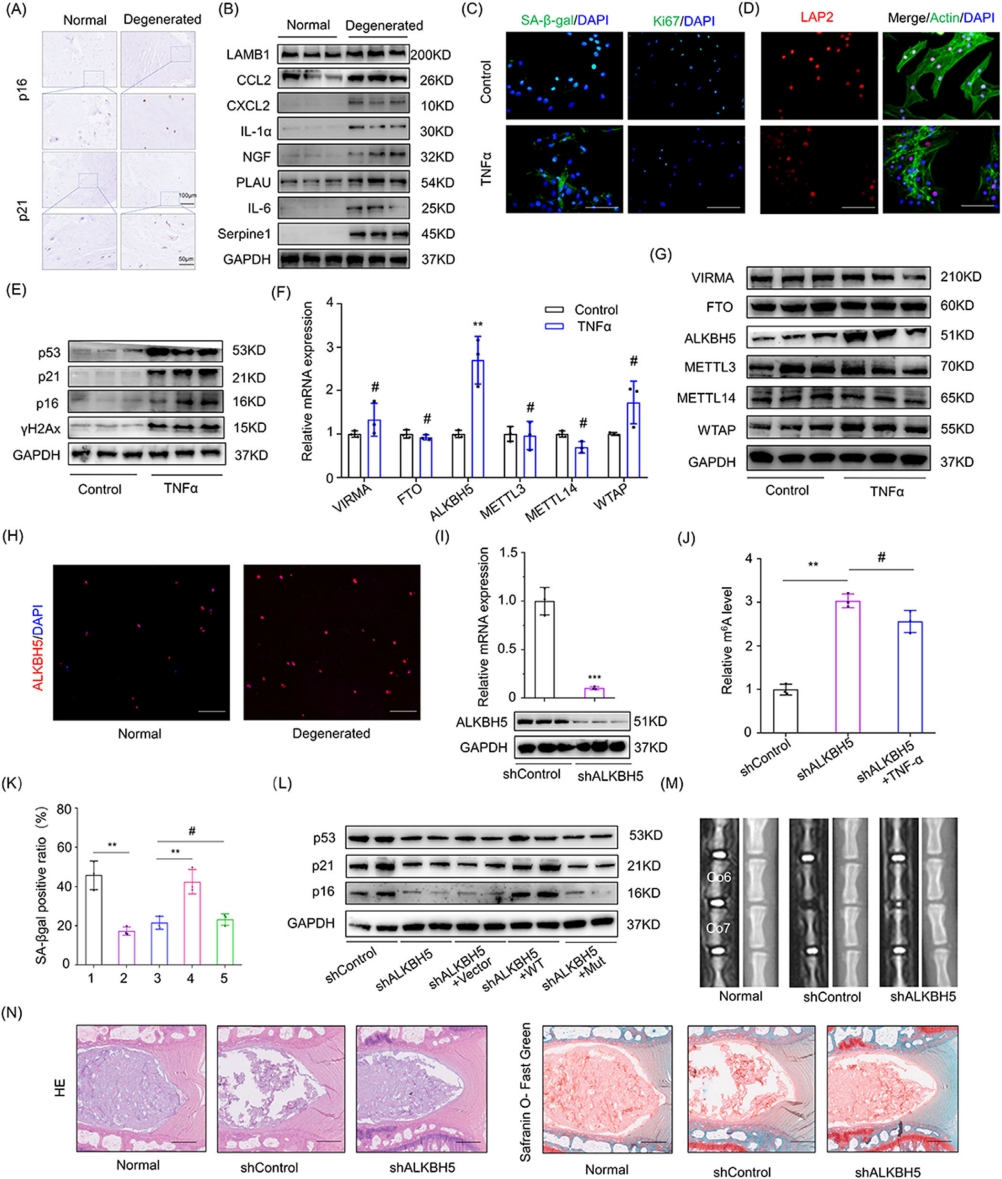


FIGURE 1 New version

We apologize for this error.

