# Supplementary material for: m6A hypomethylation of DNMT3B regulated by ALKBH5 promotes intervertebral disc degeneration via E4F1 deficiency
Source: Clin Transl Med. 2022 Mar 27;12(3):e765. doi: 10.1002/ctm2.765 (PMC8957938; doi:10.1002/ctm2.765)
Supplement: Supplementary file 1 — Supporting information [file CTM2-12-e765-s001.docx]

**SUPPLEMENTARY MATERIAL**

**Materials and Methods**

**RT-qPCR**

TRIzol reagent (Invitrogen) was used to extract total RNA from the cultured cells. The extracted RNA was reverse-transcribed using cDNA Synthesis Kit (Vazyme, NanJing, R312-01) according to the manufacturer’s instructions followed by RT-qPCR using RT SuperMix for qPCR (Vazyme, NanJing, R323-01). The primers used for RT-qPCR were listed in **Table.S2**.

**Me-RIP-PCR**

A previously described procedure was used for methylation RIP (Me-RIP) (*doi:10.1038/nprot.2015.076*). The Dynabeads mRNA Purification Kit (Invitrogen) was used to purify mRNA from the total RNA. Magna MeRIP™ m^6^A Kit (Millipore) was used to measure the change of m^6^A levels in mRNA according to the manufacturer’s protocol after RNA quality analyzing by NanoDrop 2000. After saving 0.5μg of the mRNA as input, the remaining mRNA was used for m^6^A immunoprecipitation. After immunoprecipitation with Magna ChIP protein A/G Magnetic Beads and eluted twice with elution buffer, immunoprecipitated m^6^A RNAs were recovered by ethanol precipitation, and the RNA concentration was measured with NanoDrop 2000. Then immunoprecipitated m^6^A RNA was used as templates in RT-qPCR, as described above. The primers used were listed in supplementary **Table. S2**. The housekeeping gene HPRT1 was chosen as internal control since HPRT1 mRNA does not have m^6^A peaks in m^6^A profiling.

**Immunohistochemistry (IHC)**

For IHC, paraffin-embedded tissue sections were deparaffinized with xylene and rehydrated with an alcohol gradient and water. Sections were incubated with primary antibodies p16 (diluted 1:500, CST, catalogue number: #80772), p21 (diluted 1:100, CST, catalogue number: #2947) at room temperature for 1h and biotin-labelled secondary antibodies for 30min, and then stained with Vectastain ABC kit and DAB peroxidase substrate kit (Boster, AR1000).

**Immunofluorescence Staining**

Immunofluorescence analysis was performed as following described. First, 4% paraformaldehyde was used to fix NPCs, and then 0.5% Triton X-100 in PBS was used to permeabilize. The slides were washed in PBS and blocked with 2% bovine serum albumin (BSA) in PBS for 2 h at 37 °C, and then incubated with anti-Ki67 (1:400, #9449, CST), anti-LAP2 (1:100, 14651-1-AP, Proteintech), anti-E4F1 (1:500, PA5-67167, Thermofisher) for 10 h. After washing twice, the slides were then incubated with goat anti-rabbit antibody (CST) at 37 °C for 1 h. Nuclei were then co-stained with 0.1 g/ml DAPI (Beyotime, Nantong, China), and images were captured under a microscope (Olympus, BX53; Melville, NY, USA).

**RNA Stability Assay**

After treatment with 5μg/ml actinomycin D (MedChem Express) to inhibit mRNA transcription, cells were collected at 0, 2, 4, 6 and 8 h to analyze mRNA levels and the rate of degradation. The total RNA was extracted and used for RT-qPCR. The degradation rate of RNA (k) was calculated using the equation: e^-kt^ =N_0_/N_t_, t means the time after transcription inhibition and k represents the degradation rate, and N_t_ and N0 are the relative mRNA expression at time t and time 0. The RNA half-lifetime(t_1/2_) was calculated from the degradation rate as t_1/2_=ln2/k.

**Dot Blot and m^6^A Level Quantification Assay**

Equal mRNA of separate group UV crosslinked at 254 nm after being loaded to the membrane, and then membrane was blocked with 5% nonfat dry milk (in 1X TBS) for 1 hours and incubated with anti-m^6^A antibody (1:1000, CST, #56593) at 4 ^o^C overnight. HRP-conjugated Affinipure Goat Anti-Rabbit IgG (SA00001-2, Proteintech,1:10000) was added. Protein expression was visualized using enhanced chemiluminescence reagents (Affinity, KF001, Nanjing, China) and the ChemiDoc MP Imaging System (Bio-Rad, 12003154 Hercules, CA, USA). For m^6^A quantification, Elisa-based m^6^A colorimetric assay was performed to measure m^6^A level in mRNA using EpiQuik m6A RNA Methylation Quantification Kit (Colorimetric) (Epigentek) following the manufacturer’s protocol.

**SA-β-galactosidase Staining**

NPCs were first fixed in 2% formaldehyde and 0.2% glutaraldehyde at room temperature for 5 mins after corresponding disposal and then stained using fresh staining solution at 37°C for 12 hours. Images were captured under a microscope (Olympus, BX53; Melville, NY, USA) and SA-β-gal-positive cells were counted using flow cytometry (BD FACS Calibur; BD Biosciences, San Jose, CA, USA).

**Immunoprecipitation Studies and Western Blot Analysis**

Control cells or cells transfected with expression plasmids were lysed in HEPES lysis buffer after corresponding disposal (180 mM NaCl, 1.5 mM MgCl2, 5 mM EDTA, 50 mM HEPES, 1% NP40, 10% glycerol, 0.1% sodium orthovanadate, and a mixture of protease inhibitors from Roche Applied Science). Lysates were immunoprecipitated (IP) with beads conjugated with antibodies. The associated proteins were separated on SDS–PAGE and then incubated with the specific primary antibody and visualized using enhanced chemiluminescence reagents (Affinity, KF001, Nanjing, China) and the ChemiDoc MP Imaging System (Bio-Rad, 12003154 Hercules, CA, USA).

**Chromatin Immunoprecipitation Assays (ChIP)**

Cells were conducted with corresponding disposal. ChIP was conducted using a Simple ChIP Enzymatic Chromatin IP kit (Cell Signaling Technology), according to the manufacturer’s instructions. Antibodies of anti-H3K27me3, anti-H3K4me3, anti-H3K9me3, anti-H3K16ac, anti-KDM4a, anti-Pol Ⅱ(1:30; ab238146; Abcam) were used for immunoprecipitation. Normal rabbit IgG was used as a negative control. Primers of ALKBH5, E4F1 for ChIP-qPCR are listed in **Table S2**.

**Subcellular fractionation for RT-qPCR**

RNA extraction was performed using PARIS™ kit (Invitrogen™: AM1921) and real-time PCR were performed according to the protocol of instructions, GAPDH was used as endogenous control for the cytoplasmic RNA, while 18S RNA was selected as endogenous control for the nuclear RNA. And GAPDH and H3 were using a loading protein control.

**DNA-Pulldown assays**

Two hundred million NPCs were harvested After washing once with cold PBS buffer containing inhibitors (PBSI, 0.5mM PMSF, 25mMb-glycerophosphate, 10mM NaF), cell pellets were then lysed with 300mL of buffer A (10mM HEPES, pH7.9, 1.5mM MgCl_2_, 10mM KCl, 300mM sucrose, 0.5% NP-40). Following incubated on ice for 10 min, the lysates were centrifuged at 2,600 g for 30 s and sonicated with 150mL of buffer B (20mM HEPES, pH7.9, 1.5mM MgCl_2_, 420mM NaCl, 0.2mM EDTA, 2.5% glycerol). The samples were centrifuged at 15000 rpm for 5 min, and the supernatants were incubated with 8μg of biotinylated DNA overnight at 4 ^o^C. Anti-biotin beads were used to incubate with samples for 2 to 4 h at 4 ^o^C. The pull-down proteins were eluted for western blot.

**RNA stability assay**

After treatment with 5μg/ml actinomycin D (MedChem Express) to inhibit mRNA transcription, cells were collected at 0, 2, 4, 6 and 8 h to analyze mRNA levels and the rate of degradation. The total RNA was extracted and used for RT-qPCR. The degradation rate of RNA (k) was calculated using the equation: e^-kt^ =N_0_/N_t_, t means the time after transcription inhibition and k represents the degradation rate, and Nt and N0 are the relative mRNA expression at time t and time 0. The RNA half-lifetime(t_1/2_) was calculated from the degradation rate as t_1/2_=ln2/k.

**Methylation-specific PCR (MSP) analysis**

MSP was performed with genomic DNA from NPCs with corresponding disposal. For E4F1 promoter, we used methylated forward primer: GAAGTTTAGGTCGA AGTCGG; reverse primer: AACCCGAAATAAACTAC CCG, which amplify the 2162/2328 locus relative to the transcription starting site and unmethylated forward primer: TAGAAGTTTAGGTTGAAGTTGGG and reverse primer: CAAAATAAAC TACCCACACCCC, which amplify the 2160/2324 locus relative to the transcription starting site. Input was performed as internal control after the genomic DNA was PCR-amplified with forward primer CCAACTCCAAATCCCCTCTCTAT and reverse primer TGATTAATTTAGATTGGGTTTAGAGAAGGA simultaneously. Then 2% agarose gel was used to analyze the PCR products and visualization was realized under ultraviolet light. The relative levels of PCR products were first normalized with input PCR and then presented as ratio of methylated or unmethylated PCR over total PCR products.

**RNA Interference and Plasmid Transfection**

Knockdown of ALKBH5, KDM4A, YTHDF2, DNMT3B, and E4F1 in NPCs was realized by transfection with siRNA. siRNA against ALKBH5 (siALKBH5), KDM4A (siKDM4A), YTHDF2 (siYTHDF2), DNMT3B (siDNMT3B), and E4F1 (siE4F1) and scrambled siRNA (siControl) were synthesized by Sangon Biotech (Shanghai, China) and transfected with Lipofectamine 2000 (Invitrogen) according to the standard protocol. The siRNA sequences were listed in **Table S3**. After verified high silencing efficiency, the NP cells were then used in following treatment. Vectors of lentivirus pLKO.1 were used in which shRNA against ALKBH5 (*Rat*) and DNMT3B (*Rat*) were cloned. And the targeted sequences of shRNA were listed in the Supplementary Material Table S3. And the psiCHECKTM-2 DNMT3B, pGL3-Basic-DNMT3B and pRL-TK plasmids using for dual luciferase analysis were constructed by TsingKe Biological Technology (Beijing, China). Plasmids pCDH-ALKBH5, pCDH-ALKBH5-H204A, pCDH-KDM4A, pCDH-YTHDF2, pCDH-YTHDF2-N, pCDH- YTHDF2-C, pCDH- YTHDF2-WA, pCDH-E4F1 were constructed by TsingKe Biological Technology (Beijing, China) and lentivirus pLKO.1 were used to clone.

**Luciferase Reporter Assay**

NP cells were seeded in triplicate in 24-well plates to allow 70% confluency in the next day. 100 ng reporter plasmids with DNMT3B 3’-UTR region (psiCHECKTM-2 DNMT3B) or plasmids with promoter of DNMT3B (pGL3-Basic- DNMT3B) and vector 20 ng renilla luciferase (Rluc) control plasmids (pRL-TK) were co-transfected with or without WTAP knockdown using Fugene HD (Promega #E231A). Fluc and Rluc activities were measured 24h later with the Dual-Luciferase Reporter Assay System (Promega) according to the instructions. The relative luciferase activity was calculated by dividing Fluc by Rluc and normalized to individual control for each assay.

**DNase I Sensitivity Analysis**

Nucleus of NP cells stimulated with TNFα 48 h were pretreated with DNase I (0.1 U/μl, Sigma) at 37°C for 20 min and then stopped by EDTA (50mM/ml). Genome DNA was extracted and subjected to quantitative RT -PCR assay for detection of ALKBH5 and E4F1 promoter region using ChIP primers.

**EdU Incorperation Assay**

EdU labeling was performed to examine the proliferation status of NPCs. BMSCs were exposed to 25×10^−6^ M of 5-ethynyl-2′-deoxyuridine (EdU, RiboBio, C10338, Guangzhou, China) for 2h at 37°C and fixed in 4% paraformaldehyde. NPCs were then permeabilized using 0.5% Triton-X-100 and then reacted with Apollo488 for 30 mins. subsequently, Hoechst 33342 was used to stain the DNA contents of the cells for 30 min, and images were visualized and captured using a microscope (Olympus, BX53). EdU positive cells were analyzed using Image J. The experiments were replicated three times.

**Animal Model of Intradiscal Injection**

Protocols were approved by The Institutional Animal Care and Use Committee (IACUC) at Tongji Medical College, Huazhong University of Science and Technology (NO. S2394). The surgical procedure was performed as described previously (DOI:10.1016/j.redox.2018.09.006; DOI 10.1097/BRS.0b013e31817c64a9). Total of 120 rats were used for animal modelling, with 9 rats per group in this study. After 200g male rats were anesthetized with 0.4ml of 3% (w/v) pentobarbital and grouped randomly, investigators blinded to the group allocation performed the experiments. The disc levels in rat tail (Co6/7) were located by palpation on the coccygeal vertebrae and confirmed by trial radiography. Needles (29-G) were used to puncture the annulus fibrosus layer though the tail skin, in parallel to the end plates. To ensure that the needle did not penetrate too deeply, the length of the needle was pre-determined according to the dimensions of annulus fibrosus and the NP, which were measured in a preliminary experiment and found to be approximately 4 mm. Six kinds of solution were prepared for intradisc injection, including PBS containing lentivirus of shControl, PBS containing lentivirus of shALKBH5, and PBS containing lentivirus of shDNMT3B. Each segment was injected with 2μl of the solution of interest, and each needle were kept in the disc for 10 s. All animals were allowed free, unrestricted weight bearing and activity. The injections were conducted every two weeks for one month and then keep for one month.

**Histological and Radiographic Evaluation and Analysis**

Two months later, rats were sacrificed and histological and radiographic evaluation were performed. After X-ray and MRI examination, tails were fixed in 10% neutral-buffered formalin for 1 week and Midsagittal sections were stained with hematoxylin, eosin and Safranin O-fast green while IF staining of p16 and Collagen Ⅱ was performed to evaluate the degeneration level. The histological evaluation was performed according to histologic grading system developed by Ji et al (*doi:10.1038/s41467-018-07360-1*). More specifically, the cellularity and morphology of the AF, NP, and the border between the two structures were examined. The scale is based on 5 categories of degenerative changes with scores ranging from 0 points (0 in each category) for a normal disc to 15 points (3 in each category) for a severely degenerated disc. For morphology of the NP, score 0: round shape and the NP constitutes >75% of the disc area, score 1: round shape and the NP constitutes 50–75% of the disc area, score 2: round shape and the NP constitutes 25–50% of the disc area, score 3: round shape and the NP constitutes <25% of the disc area. For cellularity of the NP, score 0: stellar-shaped cells with a proteoglycan matrix located at the periphery, evenly distributed, score 1: partially stellar and partially round cells, more stellar than round, score 2: mostly large, round cells, separated by dense areas of proteoglycan matrix, score 3: large, round cells, separated by dense areas of proteoglycan matrix. For morphology of the AF, score 0: well-organized collagen lamellae with no ruptures, score 1: inward bulging, ruptured, or serpentinefibers constitute <25% of the AF, score 2: inward bulging, ruptured, or serpentinefibers constitute 25−50% of the AF, score 3: inward bulging, ruptured, or serpentinefibers constitute >50% of the AF. For cellularity of the AF, score 0: fibroblasts comprise >90% of the cells, score 1: fibroblasts comprise>75–90% of the cells, score 2: intermediate, score 3: chondrocytes comprise >75% of the cells. For border between the NP and AF, score 0: normal, without any interruption, score 1: minimal interruption, score 2: moderate interruption, score 3: severe interruption. Radiographs were taken at 6 and 12 weeks after the puncture. The change in IVD height was evaluated by the disc height index (DHI) The change in IVD height was evaluated by the disc height index (DHI) Measurements of internal control discs were carried out together with their corresponding punctured discs. Disc height and the adjacent vertebral body heights were measured on the midline and 25% of the disc’s width from the midline on either side. The DHI was expressed as the mean of the 3 measurements from midline to the boundary of the central 50% of disc width divided by the mean of the 2 adjacent vertebral body heights. Changes in the DHI of punctured discs were expressed as a percentage (%DHI=post-punctured DHI/pre-punctured DHI ×100).

**Supplementary Figures**

**
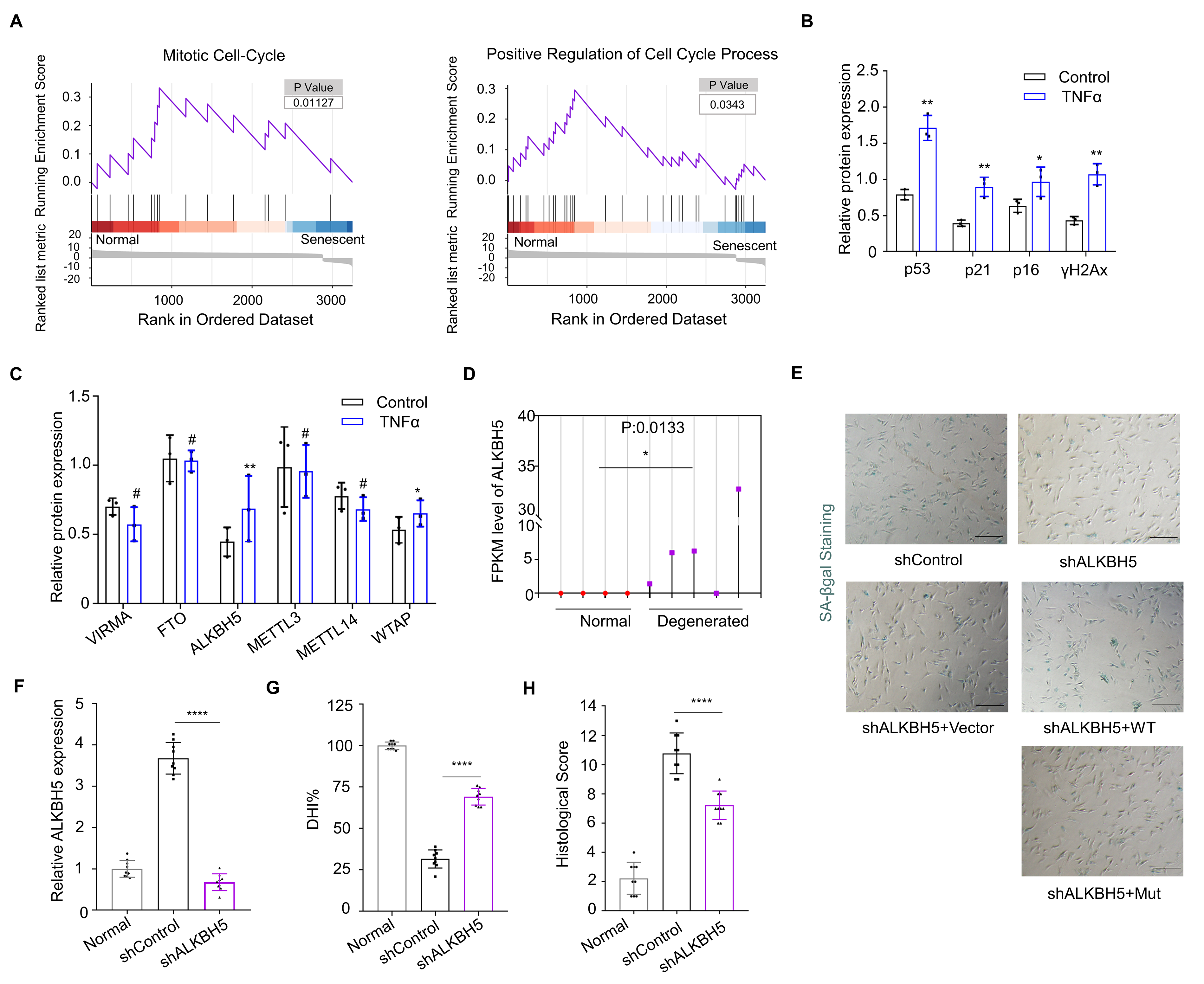
**

**Figure S1.**

(**A**) GSEA results showed that degeneration of NP was accompanied by NPC cell cycle block.

(**B**) Protein level analysis of senescence indicators in normal and senescent NPCs by western blot, GAPDH was used as a loading control, n = 3. **P* < 0.05, ***P* <0.01, two-tailed unpaired Student’s t-test.

(**C**) Protein level analysis of methyltransferases and demethylases in normal and senescent NPCs by western blot, GAPDH was used as a loading control, n = 3. **P* < 0.05, ***P* < 0.01, ^#^*P* > 0.05, two-tailed unpaired Student’s t-test.

(**D**) Analysis of ALKBH5 FPKM in the transcript sequencing data of NPCs from degenerated and normal discs. **P* < 0.05.

(**E**) SA-β-gal activity staining (scale bar: 50 μm) and analysis of NPCs with ALKBH5 silencing accompanied with overexpression of wild-type or mutant ALKBH5.

(**F**) Relative expression of ALKBH5 in NP tissues of Rats with different disposal by RT-qPCR. GAPDH was used as a loading control. n=9. ****P*<0.001, two-tailed unpaired Student’s t-test. *Control* represents group without surgery, *shControl* represents group with surgery using lentivirus containing shControl, *shALKBH5* represents group with surgery using lentivirus containing shALKBH5.

(**G, H**) Radiographic imaging analysis and histological staining of a rat model of IVD degeneration (scale bar: 500 μm) with or without ALKBH5 silencing, n = 9. *****P* < 0.0001, two-tailed unpaired Student’s t-test.

**
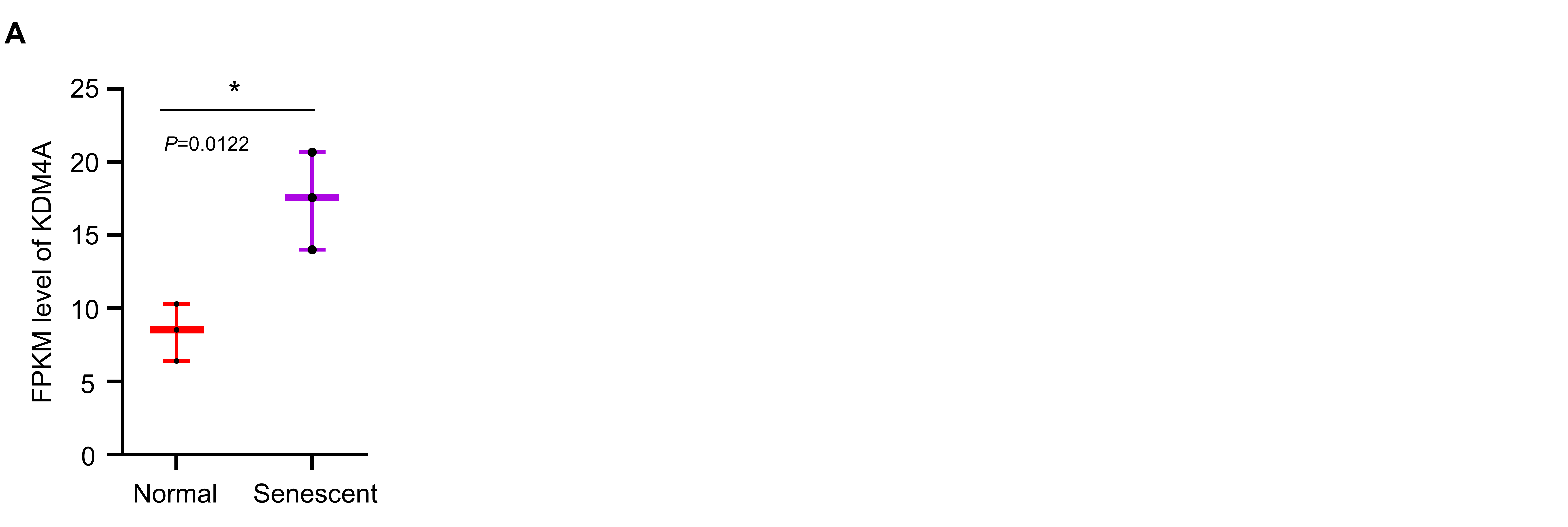
**

**Figure S2.**

1. Analysis of KDM4A FPKM in the transcript sequencing data of normal and senescent NPCs, n=3, **P* < 0.05, two-tailed unpaired Student’s t-test.

**
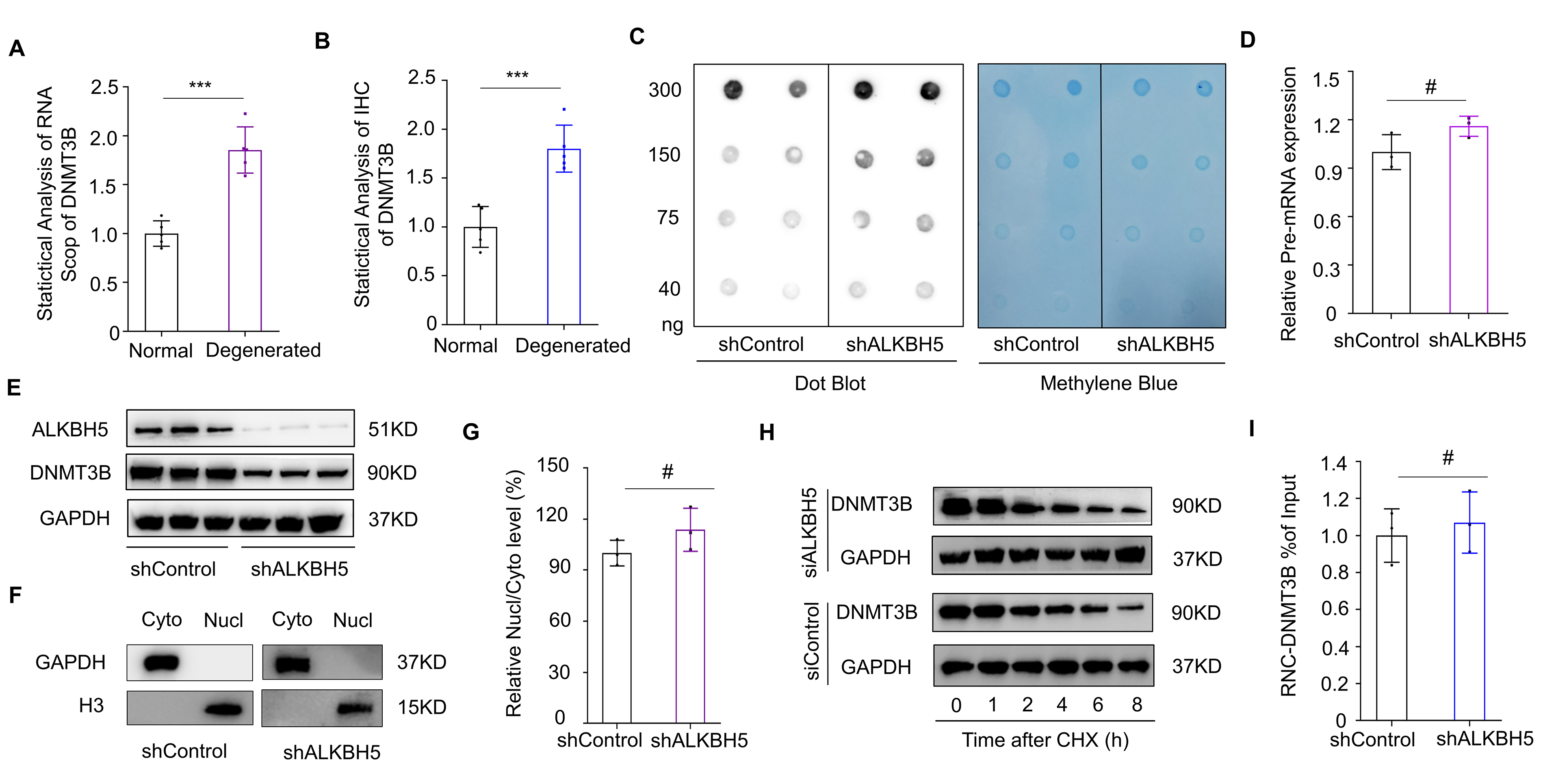
**

**Figure S3**.

(**A**) Statistical analysis of DNMT3B mRNA expression of RNA Scop, n=5. ****P* < 0.001, two-tailed unpaired Student’s t-test.

(**B**) Statistical analysis of DNMT3B protein expression of IHC, n=5. ****P* < 0.001, two-tailed unpaired Student’s t-test.

(**C**) Dot blot analysis (left) of global m6A modification of NPCs with or without ALKBH5 silencing. Methylene blue staining (right) of equal mRNA was using as loading control.

(**D**) Expression of pre-mRNA of DNMT3B in NPCs with ALKBH5 silencing or not by RT-qPCR, GAPDH was used as a loading control. n=3. ^#^*P*>0.05, two-tailed unpaired Student’s t-test.

(**E**) Expression of ALKBH5 and DNMT3B in NPCs with or without ALKBH5 silencing by western blot analysis, GAPDH was used as a loading control, n = 3.

(**F, G**) Subcellular localization analysis of DNMT3B mRNAs using RT-qPCR by separating RNAs in nuclear and cytoplasm. n=3. ^#^*P*>0.05, two-tailed unpaired Student’s t-test.

(**H**) Protein stability analysis of DNMT3B by western blot in NPCs with ALKBH5 silencing or not after CHX treatment.

(**I**) Ribosome-nascent chain complex qPCR (RNC-qPCR) to assess the translational efficiency of DNMT3B, n = 3. ^#^*P* > 0.05, two-tailed unpaired Student’s t-test.

**
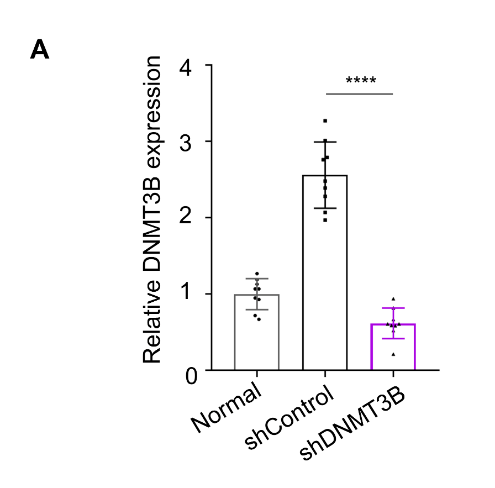
**

**Figure S4.**

(**A**) Relative expression of DNMT3B in NP tissues of Rats with different disposal by RT-qPCR. GAPDH was used as a loading control. n=9. *****P*<0.0001, two-tailed unpaired Student’s t-test.

**Supplementary Tables**

**Table S1.** **Antibodies used in this study**

| **Product name** | **Catalog No.** | **Manufacturer** | **Dilution Ratio** | **Dilution Ratio** |
| --- | --- | --- | --- | --- |
| anti-p21 | #2947 | CST | 1:1000 | Western Blot |
| anti-p16 | #80772 | CST | 1:1000 |  |
| anti-p53 | #2527 | CST | 1:1000 |  |
| anti-METTL3 | ab195352 | Abcam | 1:1000 |  |
| anti-VIRMA | 25712-1-AP | Proteintech | 1:500 |  |
| anti-METTL14 | ab220030 | Abcam | 1:1000 |  |
| anti-WTAP | ab195380 | Abcam | 1:1000 |  |
| anti-FTO | ab126605 | Abcam | 1:1000 |  |
| anti-ALKBH5 | ab195377 | Abcam | 1:1000 |  |
| anti-GAPDH | 60004-1-Ig | Proteintech | 1:10000 |  |
| anti-H3 | 17168-1-AP | Proteintech | 1:5000 |  |
| anti-H3K9me3 | ab8898 | Abcam | 1:1000 |  |
| anti-KMT1A | ab245380 | Abcam | 1:1000 |  |
| anti-KDM4A | ab191433 | Abcam | 1:1000 |  |
| anti-IGF2BP1 | 22803-1-AP | Proteintech | 1:500 |  |
| anti- IGF2BP2 | 11601-1-AP | Proteintech | 1:1000 |  |
| anti- IGF2BP3 | 14642-1-AP | Proteintech | 1:1000 |  |
| anti-YTHDF1 | 17479-1-AP | Proteintech | 1:1000 |  |
| anti-YTHDF2 | 24744-1-AP | Proteintech | 1:5000 |  |
| anti-LAMB1 | ab256380 | Abcam | 1:500 |  |
| anti-CCL2 | ab200343 | Abcam | 1:500 |  |
| anti-E4F1 | ab70615 | Abcam | 1:1000 |  |
| anti-CXCL2 | ab275879 | Abcam | 1:500 |  |
| anti-IL1α | ab254360 | Abcam | 1:500 |  |
| anti-NGF | # 2046S | CST | 1:1000 |  |
| anti-PLAU | 17968-1-AP | Proteintech | 1:1000 |  |
| anti-γH2AX | ab81299 | Abcam | 1:1000 |  |
| anti-IL6 | 21865-1-AP | Proteintech | 1:1000 |  |
| anti-Serpine1 | 66261-1-Ig | Proteintech | 1:1000 |  |
| anti- m^6^A | #56593 | CST | 1:100 | RIP |
| anti-YTHDF2 | 24744-1-AP | Proteintech | 1:20 |  |
| anti-DNMT3B | #57868 | CST | 1:1000 | IF |
| anti-LAP2 | ab185718 | Abcam | 1:500 |  |
| anti-Ki67 | #9449 | CST | 1:1000 |  |
| anti-Colla Ⅱ | 28459-1-AP | Proteintech | 1:800 |  |
| anti-H4K16ac | ab109463 | Abcam | 1:1000 | ChIP |
| anti-H3K4me3 | ab213224 | Abcam | 1:1000 |  |
| anti-H3K9me3 | ab8898 | Abcam | 1:1000 |  |
| anti-H3K27me3 | ab6002 | Abcam | 1:1000 |  |
| anti-Pol 2 | ab264350 | Abcam | 1:20 |  |
| anti-DNMT3B | #57868 | CST | 1:1000 |  |

**Tables S2*.* Primers sequences**

| Homo VIRMA | Forward | 5′- AGCGAAGTCCCGAACGACGA -3′ |
| --- | --- | --- |
|  | Reverse | 5′- TGGGCATTTCCAACGGGCCAA -3′ |
| Homo METTL3 | Forward | 5′- TTTTCCGGTTAGCCTTCGGG -3′ |
|  | Reverse | 5′- GATAGAGCTCCACGTGTCCG -3′ |
| Homo METTL14 | Forward | 5′- AATGGCCGTTCTGTGCTCAT -3′ |
|  | Reverse | 5′- AAGGACCCATCACAGGCAAG -3′ |
| Homo WTAP | Forward | 5′- GAAAGGACGGGGAGTGTTAC -3′ |
|  | Reverse | 5′- CCCACCACAGCTTTCTCGTA -3′ |
| Homo ALKBH5 | Forward | 5′- TGCAAGCTCATGCAAACACC -3′ |
|  | Reverse | 5′- CCCCCAAAGTGGTGGTATCC -3′ |
| Homo FTO | Forward | 5′- CGCATGGCAGCAAGCTAAAT -3′ |
|  | Reverse | 5′- TGCACATTCCCTGACTCCAC -3′ |
| Homo DNMT3B | Forward | 5′- CCGCTTCCTCGCAGCAG -3′ |
|  | Reverse | 5′- TGGGCTTTCTGAACGAGTCC -3′ |
| Homo Pre-DNMT3B | Forward | 5′- CACTGAGCAATTCCCCAAACG -3′ |
|  | Reverse | 5′- TCCCAGTACTGTGCTTTTGCT -3′ |
| Homo ALKBH5-ChIP | Forward | 5′- ACTGCCTGATTGACACGCAT -3′ |
|  | Reverse | 5′- CCTTTGGCGCTTCCACTTCT -3′ |
| Homo ALKBH5-ChIP-Pol 2 | Forward | 5′- GGGGTTCGGCGCTAAGG -3′ |
|  | Reverse | 5′- CTTGAGCTTCTCACGCAGGTC-3′ |
| Homo E4F1-ChIP | Forward | 5′- GCCTTAGTAAGCAATGTAACACACA -3′ |
|  | Reverse | 5′- AGGCTCTGGATCAGGCTGTA -3′ |
| Homo HPRT1-RIP | Forward | 5′- CCCTGGCGTCGTGATTAGTG-3′ |
|  | Reverse | 5′-TCGAGCAAGACGTTCAGTCC-3′ |
| Pull down:  T7-DNMT3B | Forward: | 5′- TAATACGACTCACTATAGGGAGACCTGGAAGGTGAGCGAAGTC -3′ |
|  | Reverse: | 5-ATTTAGGTGACACTATAGAAGGGACGTGGCCTGTCATTCTACC-3’′ATTTAGGTGACACTATAGAAGGGACGTGGCCTGTCATTCTACC -3′ |
| Homo DNMT3B-RIP | Forward | 5′- CAAAACCACAGTGCCGACAG -3′ |
|  | Reverse | 5′- TTCTCTTCTGCTTGCCCTGG -3′ |

**Tables S3. siRNA or shRNA Target sequences**

| Sequence name | Target-position | Target-sequence |
| --- | --- | --- |
| Homo-siControl | NA | 5′- UUCUCCGAACGUCACGUTT -3′  5′- ACGUGACACGUUCGGAGAATT -3′ |
| Homo-siALKBH5 | 2452-2474 | 5′- UUUUCCAUACCCCAAAUGCUA -3′  5′-GCAAGUACACAGAUCUUAACU-3′ |
| Homo-siDNMT3B1 | 2419-2441 | 5′- AACUUUGAUGGCAUCAAUCAU -3′  5′-GAUCAACUAGCAAAAUUUUGG -3′ |
| Homo-siDNMT3B2 | 3071-3093 | 5′- ACUACAAAUCAUUUUUGGGAA -3′  5′- CAUCAAAAGGAUGGAUUAAAC-3′ |
| Homo-siYTHDF2 | 1875-1897 | 5′- UUUUAACACUUUCUUCUUCCU-3′  5′- GAAGAAGAAAGUGUUAAAAAG-3′ |
| Homo-shALKBH5 | 197-219 | 5′- UUUCUUUUUAUUUUUCCGCAC-3′  5′- GCGGAAAAAUAAAAAGAAAAG-3′ |
| Rat-shALKBH5 | 1320-1342 | 5′- UCUUUCACCAAUUCUGUUCUU-3′  5′- GAAGAAGAAAGUGUUAAAAAG-3′ |
| Rat-shDNMT3B | 3359-3381 | 5′- AAAUCUCAGGUGAUUUUGCAG-3′  5′- GCGGAAAAAUAAAAAGAAAAG-3′ |
